# The Myokine Landscape: Sources, Signaling, and Context-Dependent Actions

**DOI:** 10.3390/cells15181682

**Published:** 2026-09-17

**Authors:** Carme Casadevall, Alba Ramírez-Sarmiento, Ramon Camps-Ubach, Esther Barreiro, Mauricio Orozco-Levi, Joaquim Gea

**Affiliations:** 1 Respiratory Department, Hospital del Mar Research Institute (HMRIB), Hospital del Mar, 08003 Barcelona, Spain; 2 MELIS Department, Universitat Pompeu Fabra, 08003 Barcelona, Spain; 3 Area of Respiratory Diseases (CIBERES), CIBER, ISCiii, 28029 Madrid, Spain; 4 Respiratory Department, Hospital Internacional de Colombia, Fundación Cardiovascular de Colombia, Bucaramanga 680001, Colombia; 5 Department of Medicine, Universidad de Santander (UDES), Bucaramanga 680003, Colombia

**Keywords:** myokines, skeletal muscle, exercise, muscle regeneration, muscle secretome

## Abstract

In addition to its contractile role, skeletal muscle has important secretory functions and communicates with local and distant tissues through myokines, a heterogeneous group of cytokines, chemokines, growth factors, peptides, and extracellular-matrix-associated proteins released by muscle cells. Their production is regulated by contraction, mechanical loading, energy availability, hypoxia, inflammation, injury, and mitochondrial or metabolic stress. Through autocrine, paracrine, and endocrine mechanisms, myokines influence satellite-cell activity, myogenesis, protein turnover, muscle mass, metabolism, mitochondrial function, angiogenesis, immune-cell recruitment, extracellular-matrix remodeling, and inter-organ communication. This review provides a structured overview of current knowledge on the production, regulation, and biological actions of approximately 25 major myokines, with particular emphasis on the strength of evidence supporting their classification as bona fide muscle-derived factors. We distinguish local muscle signaling from changes in circulating concentrations of uncertain tissue origin and highlight the context-dependent nature of myokine actions. The same mediator may support adaptation and repair when transiently and locally produced but contribute to inflammation, metabolic dysfunction, fibrosis, or muscle wasting when signaling is excessive, prolonged, or disease-associated. Defining cellular sources, temporal regulation, receptor availability, and interactions within the muscle secretome will be essential for clarifying physiological relevance and translating myokine biology into biomarkers and therapeutic strategies.

## 1. Introduction

Over the past three decades, the perception of skeletal muscle has expanded beyond its primary contractile and mechanical functions to include an increasingly recognized role in local and systemic signaling. The identification of signaling molecules produced by skeletal muscle in response to exercise, metabolic demand, injury, and other stimuli has led to the progressive development of the myokine concept and to recognition of a broad repertoire of muscle-derived mediators involved in tissue adaptation and inter-organ communication [1,2,3].

This rapidly expanding field has also generated important conceptual and methodological challenges. Factors currently described as myokines are supported by markedly different levels of evidence regarding their production and secretion by muscle cells, and changes in muscle expression or circulating concentrations do not necessarily establish a muscular origin. Moreover, findings obtained in cultured cells or experimental animals are not always reproduced in humans, and the biological effects of individual mediators may vary substantially according to cellular source, receptor availability, concentration, timing, and physiological or pathological context. These uncertainties complicate comparison across studies and remain particularly relevant when considering the potential use of myokines as biomarkers or therapeutic targets.

Accordingly, this narrative Review examines the established physiological actions of myokines and subsequently discusses emerging or putative functions for which the evidence remains incomplete. It then addresses how alterations in myokine production or signaling may contribute to disease, with particular emphasis on the context-dependent nature of their effects, and considers current limitations and future perspectives regarding their potential relevance as biomarkers or therapeutic targets.

The primary aim of this Review is therefore to provide a structured and accessible reference framework on the major myokines and muscle-derived factors, particularly for readers from diverse clinical and biomedical fields who may not be specialists in myokine biology, and thereby to facilitate the interpretation of the more focused contributions in this Special Issue. A secondary aim is to identify areas of mechanistic convergence, uncertainty, conflicting evidence, and ongoing debate.

### Definition and Scope of Myokines

Skeletal muscle has traditionally been regarded primarily as the organ responsible for contraction and movement, posture, and force generation in limb muscles or pressure generation in respiratory muscles. In addition to these primary functions, skeletal muscle has important secretory and signaling capabilities that enable communication with both adjacent and distant tissues [1,2]. This communication is mediated, at least in part, by myokines [1], broadly defined as signaling molecules produced and released by skeletal muscle cells that exert autocrine, paracrine, or endocrine effects [1,3]. The identification of interleukin-6 as a contraction-induced muscle-derived factor provided some of the earliest direct evidence supporting this concept and established a new paradigm in which skeletal muscle participates actively in systemic homeostasis [4]. However, the myokine category also includes non-cytokine molecules, such as irisin, myostatin/GDF-8, VEGF-A, BDNF, and extracellular-matrix-associated proteins (Table 1). Therefore, the designation “myokine” refers primarily to a factor’s production and release by skeletal muscle cells and its signaling capacity, rather than to membership in a specific molecular or functional class. Myokines should therefore be regarded as a heterogeneous group of signaling molecules that may differ markedly in molecular structure, receptor usage, target cells, and biological actions.

It should be further emphasized that myokines and cytokines are not synonymous concepts. Cytokines are signaling proteins involved in the regulation of immune and inflammatory responses, metabolism, growth, and tissue repair, and they can be produced by a wide range of cell types [5]. These include cells of the myeloid lineage, such as monocytes, blood-derived and tissue-resident macrophages, dendritic cells, neutrophils, eosinophils, basophils, and osteoclasts, among many others [5]. When cytokines are produced by myogenic cells within skeletal muscle tissue (such as muscle fibers, satellite cells, myoblasts, or myotubes) they are referred to as myokines. However, as mentioned above, many other myokines are not cytokines.

**Figure 1:**
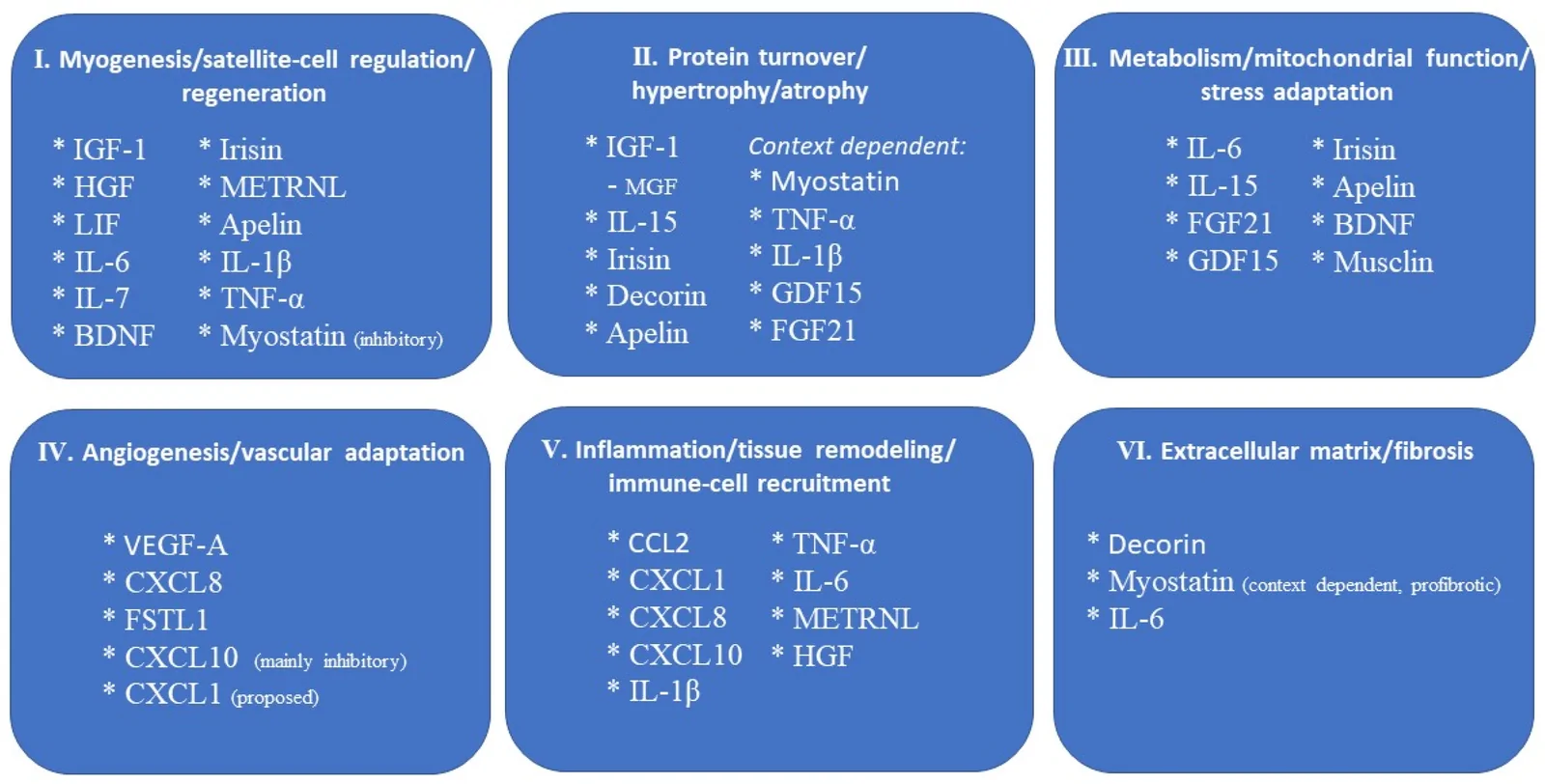
Major local actions of myokines in skeletal muscle. Schematic representation of selected myokines and muscle-derived factors grouped according to their principal reported actions within the skeletal muscle environment: myogenesis, satellite-cell regulation, and regeneration; protein turnover and regulation of muscle mass; metabolism, mitochondrial function, and stress adaptation; angiogenesis and vascular adaptation; inflammation, tissue remodeling, and immune-cell recruitment; and extracellular-matrix regulation and fibrosis. The categories are not mutually exclusive, and individual myokines may participate in several biological processes. Their effects may be stimulatory, inhibitory, or context-dependent according to their cellular source, concentration, receptor availability, duration of exposure, and physiological or pathological setting. The figure summarizes the predominant actions discussed in the text and does not imply that every listed effect has been demonstrated in humans or that each factor acts exclusively through the indicated pathway. By grouping factors according to biological actions rather than molecular identity, this figure is intended to complement the molecule-by-molecule organization of the text and to highlight functional overlap among different myokines. Detailed molecular classification and factor-specific information are provided in Table 1. BDNF, brain-derived neurotrophic factor; CCL2, C-C motif chemokine ligand 2; CXCL, C-X-C motif chemokine ligand; FGF21, fibroblast growth factor 21; FSTL1, follistatin-like protein 1; GDF15, growth differentiation factor 15; HGF, hepatocyte growth factor; IGF-1, insulin-like growth factor 1; IL, interleukin; LIF, leukemia inhibitory factor; METRNL, meteorin-like; MGF, mechano growth factor; TNF-α, tumor necrosis factor α; VEGF-A, vascular endothelial growth factor A.

**Figure 2:**
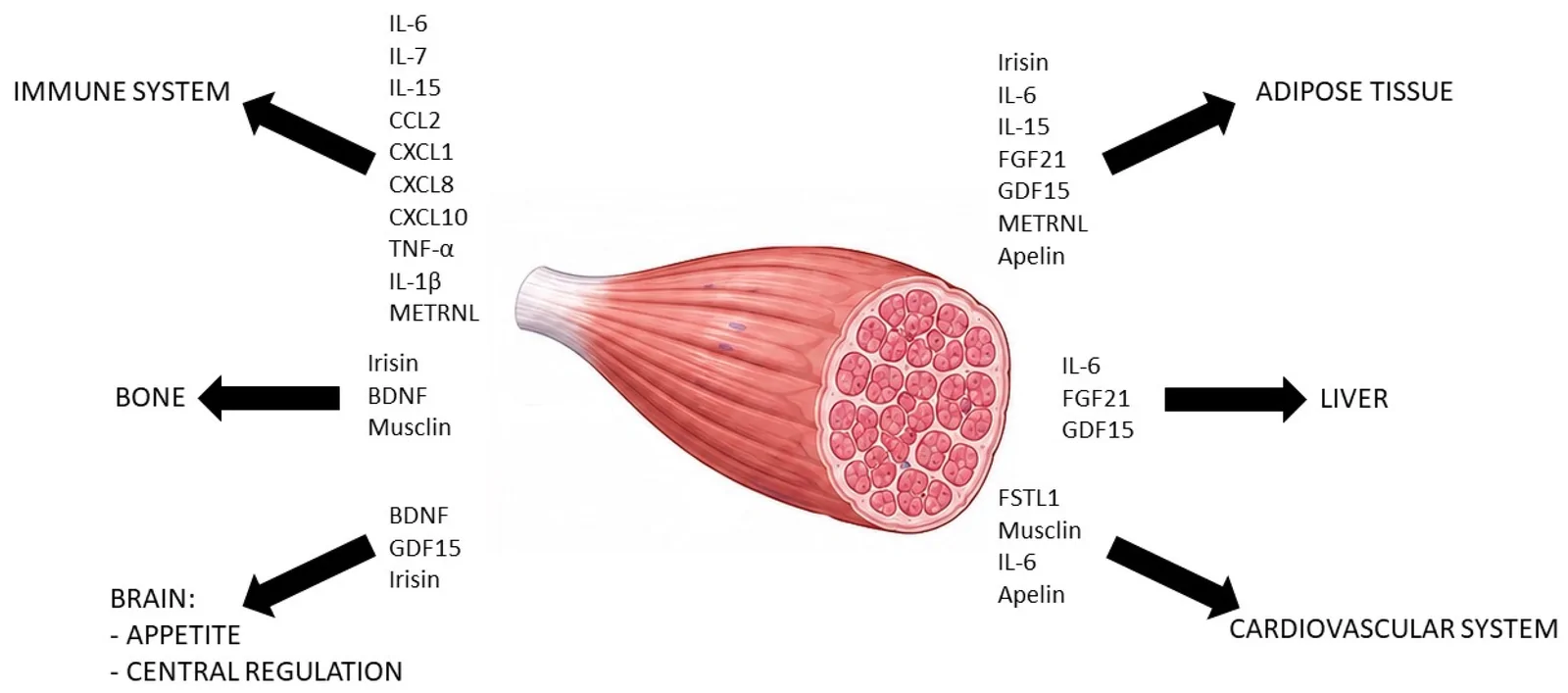
Proposed inter-organ actions of muscle-derived factors. Schematic representation of selected myokines proposed to mediate communication between skeletal muscle and adipose tissue, liver, the cardiovascular system, brain, bone, and the immune system. The arrows indicate the proposed direction of muscle-to-organ signaling but do not represent the magnitude, directness, or relative importance of each effect. The strength of the evidence varies substantially among factors and target organs, ranging from direct demonstration of muscular secretion and inter-organ signaling to associations or mechanistic observations obtained predominantly from cultured cells and animal models. Moreover, many of the factors shown are also produced by non-muscle tissues; therefore, their presence in the circulation or their effects on distant organs cannot necessarily be attributed exclusively to skeletal muscle. The figure is illustrative and not exhaustive. BDNF, brain-derived neurotrophic factor; CCL2, C-C motif chemokine ligand 2; CXCL, C-X-C motif chemokine ligand; FGF21, fibroblast growth factor 21; FSTL1, follistatin-like protein 1; GDF15, growth differentiation factor 15; IL, interleukin; METRNL, meteorin-like; TNF-α, tumor necrosis factor α.

Under physiological conditions, myokines contribute to the adaptation to exercise and to the regulation of energy metabolism, substrate availability, inflammatory responses, vascular function, tissue growth, and muscle repair (Figure 1) [6]. Through these actions, they also facilitate communication between skeletal muscle and organs such as the liver, adipose tissue, bone, the cardiovascular system, and the brain (Figure 2) [6]. Figure 1 therefore provides a functional overview of overlapping myokine actions, whereas Figure 2 illustrates proposed inter-organ communication; complementary molecule-by-molecule information on molecular class, cellular source, signaling pathways, level of evidence, and potential clinical relevance is summarized in Table 1. Nevertheless, the strength of evidence supporting these functions varies considerably among individual myokines. For some molecules, their muscular origin, secretion, target tissues, and biological effects have been demonstrated experimentally, whereas for others the available evidence remains indirect or is largely derived from cellular and animal models.

## 2. Main Myokines and Their Actions

To facilitate the use of this Review as a reference framework, the following subsections examine each factor using a broadly parallel set of criteria: evidence for production and secretion by skeletal muscle cells, regulation by physiological or pathological stimuli, receptor and major signaling pathways, local versus systemic actions, and strength and limitations of the available evidence, explicitly distinguishing findings derived from in vitro or cellular models, in vivo animal studies, and human observational or interventional studies whenever such evidence is available. A concise molecule-by-molecule summary of these different levels of evidence is also provided in Table 1. This organization is intended to allow direct comparison across factors while preserving the information required by readers less familiar with individual myokines. Because many of these molecules are also produced by non-muscle tissues, changes in muscle expression or circulating concentrations are not considered sufficient evidence of muscle-cell-derived secretion unless supported by appropriate cellular, tissue-specific, or arteriovenous data.

- ***Interleukin 6 (IL-6)***

IL-6 is a pleiotropic cytokine and the prototypical contraction-regulated myokine [2]. Skeletal muscle fibers express and release IL-6 in response to contractile activity, and studies using arteriovenous measurements have demonstrated substantial net IL-6 release from exercising human muscle. The magnitude of this response depends on the duration and intensity of exercise, the amount of muscle mass recruited, and the availability of metabolic substrates [7,8]. In particular, low intramuscular glycogen availability markedly increases IL-6 transcription and release, supporting the concept that IL-6 acts as a signal of muscular energy demand. Lactate accumulation and associated changes in intracellular pH may also promote IL-6 release through activation of intramuscular protease activity [9,10]. During physiological exercise, myofibers constitute an important source of IL-6, and its release can occur in the absence of substantial muscle damage or a preceding increase in tumor necrosis factor α. Nevertheless, skeletal muscle tissue contains several other potential IL-6-producing cells, including satellite cells, macrophages, endothelial cells, and fibro-adipogenic or stromal cells. Their relative contribution becomes particularly relevant following muscle injury or in chronic inflammatory disease. Thus, the detection of IL-6 in a muscle biopsy does not, by itself, demonstrate its production by muscle fibers. IL-6 signaling is initiated through a receptor complex containing the IL-6 receptor α subunit and the common signal-transducing protein gp130 [11]. In classical signaling, IL-6 binds to membrane-associated IL-6 receptor α, whereas in trans-signaling it binds to a soluble form of this receptor, allowing the complex to activate gp130 on cells that do not express membrane IL-6 receptor α. Receptor activation primarily engages the Janus kinase/signal transducer and activator of transcription 3 (JAK/STAT3) pathway. The biological consequences depend on receptor availability, the responding cell type, and the magnitude and duration of IL-6 exposure. More broadly, the pleiotropic inflammatory actions of IL-6 are also signaling- and context-dependent: classical signaling is frequently associated with homeostatic, regenerative, or anti-inflammatory responses, whereas trans-signaling is more commonly linked to pro-inflammatory effects, although this distinction is not absolute and varies with the tissue environment and duration of exposure [11]. The respective contributions of classical and trans-signaling to normal human muscle adaptation remain incompletely defined. One of the main proposed functions of contraction-induced IL-6 is the regulation of muscle energy metabolism. Acute IL-6 exposure increases AMPK activity and has been shown experimentally to enhance glucose uptake and fatty-acid oxidation in muscle cells. In human skeletal muscle, IL-6 has also been shown to increase basal and insulin-stimulated glucose uptake [12,13]. These findings suggest that muscle-derived IL-6 may help match substrate utilization to the increased energy requirements of prolonged exercise. However, the magnitude of its direct contribution relative to contraction-dependent, insulin-dependent, and other metabolic pathways remains uncertain. Locally produced IL-6 also regulates satellite-cell activity and load-induced muscle adaptation. During compensatory muscle growth, IL-6 is transiently expressed by enlarging myofibers and associated satellite cells. Genetic deletion of IL-6 in mice reduces satellite-cell proliferation and blunts overload-induced hypertrophy, indicating that local IL-6 signaling is functionally required for a normal hypertrophic response in this model [14]. IL-6-dependent activation of STAT3 promotes satellite-cell expansion and progression through the myogenic lineage, partly through the induction of proliferation-associated genes such as cyclin D1 and c-Myc [14]. Human studies have similarly found associations between exercise-induced IL-6/STAT3 signaling and the subsequent satellite-cell response, although this evidence is less directly causal than that obtained from animal models. IL-6 may therefore contribute to the early proliferative phase of muscle repair, increasing the number of progenitor cells available for regeneration. It also participates in extracellular-matrix remodeling and in the recovery of muscle mass after disuse-induced atrophy. Experimental loss of IL-6 delays the initial recovery of muscle mass after unloading and alters matrix remodeling during compensatory growth [15]. Nevertheless, persistent STAT3 activation may interfere with the balance between satellite-cell expansion, differentiation, and self-renewal [16]. Effective regeneration is therefore likely to require a transient increase in local IL-6 signaling followed by its timely attenuation.

The effects of IL-6 on skeletal muscle are strongly context-dependent. Transient IL-6 production during exercise or acute tissue repair differs fundamentally from chronic systemic exposure associated with chronic inflammatory or catabolic conditions [7,16]. Sustained activation of IL-6-family cytokine signaling and JAK/STAT3 can promote muscle wasting by suppressing anabolic signaling, altering protein turnover, and activating catabolic pathways [16]. In these pathological conditions, however, IL-6 may originate predominantly from tumors, immune cells, adipose tissue, or other organs rather than predominantly from muscle fibers [17,18]. It would therefore be inappropriate to attribute the adverse consequences of chronically elevated circulating IL-6 specifically to its function as a myokine.

Overall, muscle-derived IL-6 can be regarded as a contraction-, energy-demand-, and injury-responsive myokine that acts through autocrine, paracrine, and endocrine mechanisms. Its best-established actions within skeletal muscle include regulation of substrate metabolism, satellite-cell proliferation, load-induced hypertrophy, extracellular-matrix remodeling, and early regenerative responses. These effects appear predominantly adaptive when IL-6 production is transient and locally regulated, whereas sustained systemic IL-6 signaling, which in chronic pathological conditions may originate predominantly from non-muscle sources, may contribute to muscle catabolism and impaired tissue homeostasis.

- ***Interleukin 7 (IL-7)***

IL-7 is a member of the common γ-chain cytokine family with well-established functions in lymphocyte development, survival, and homeostasis. Skeletal muscle cells can also produce and secrete IL-7, supporting its classification as a cytokine-type myokine [19,20]. IL-7 was detected in conditioned media from primary human myotubes derived from satellite cells, with its concentration increasing progressively during incubation. Its expression was confirmed at both the mRNA and protein levels, and immunofluorescence demonstrated the presence of IL-7 in multinucleated myotubes expressing myosin heavy chain. These observations provide direct evidence that differentiated human muscle cells are a cellular source of IL-7. Muscular IL-7 production appears to be regulated during myogenic differentiation and adaptation to training. IL-7 expression increased as human satellite-cell-derived myoblasts differentiated into myotubes. In addition, 11 weeks of strength training induced approximately threefold and fourfold increases in IL-7 mRNA in the vastus lateralis and trapezius muscles, respectively [19]. These findings indicate that muscular IL-7 expression is responsive to sustained mechanical loading or training adaptation. However, net release of IL-7 from exercising human muscle has not been established through arteriovenous measurements. Muscle-derived IL-7 should therefore be considered primarily a putative autocrine or paracrine mediator rather than a demonstrated endocrine exercise hormone [19,20].

IL-7 signals through a heterodimeric receptor composed of the IL-7 receptor α-chain, also known as CD127, and the common cytokine receptor γ-chain, or CD132. Receptor activation involves the Janus kinases JAK1 and JAK3 and results prominently in activation of STAT5-dependent transcription [21]. In human myogenic cells, expression of the IL-7 receptor was substantially higher in satellite cells than in differentiated myotubes, with receptor mRNA decreasing by approximately 80% during differentiation. This expression pattern suggests that undifferentiated muscle progenitor cells may be more responsive to IL-7 than mature myotubes [19].

The best-described direct action of IL-7 in skeletal muscle is the regulation of myogenic-cell development. Exposure of differentiating human muscle cells to recombinant IL-7 reduced the expression of the terminal differentiation markers myogenin and myosin heavy chain (MYHC) 2 by approximately 35%. IL-7 also increased satellite-cell migration by approximately 40% after 48 h but did not significantly alter their proliferation [19]. These findings suggest that IL-7 may maintain myogenic progenitors in a relatively undifferentiated and migratory state, potentially facilitating their movement toward areas requiring growth or repair before terminal differentiation occurs. This proposed function differs from that of myokines such as LIF or IL-6 [22,23]. Current evidence does not establish that IL-7 is essential for muscle hypertrophy, regeneration, or recovery after injury. Nor has a temporal sequence been demonstrated in which an early rise in IL-7 promotes progenitor-cell migration and its subsequent attenuation permits differentiation and fusion. Such a model is biologically plausible on the basis of the available cellular data but remains hypothetical [19]. Muscle-derived IL-7 could also provide a link between skeletal muscle and the immune system. IL-7 is a major regulator of T-cell survival and homeostasis, raising the possibility that sustained muscular production contributes locally or systemically to immune regulation. However, the quantitative contribution of skeletal muscle to circulating IL-7 has not been established, and stromal tissues are important physiological sources of this cytokine [21].

The pathological relevance of muscular IL-7 also remains poorly characterized. Alterations in IL-7 production or receptor expression could theoretically affect the availability, migration, or differentiation of muscle progenitor cells during aging, chronic inflammation, muscular disease, or impaired regeneration. However, there is currently insufficient evidence to determine whether muscle-derived IL-7 is protective, maladaptive, or merely associated with these conditions. Similarly, persistent systemic IL-7 exposure may have immunological consequences that are distinct from those of transient, locally produced IL-7 within the muscle microenvironment [20,21].

Overall, muscle-derived IL-7 can be regarded as a training-responsive myokine with putative autocrine or paracrine actions and a potential role in the regulation of myogenic-cell development. Its best-supported actions include enhancement of satellite-cell migration and reduced terminal myogenic differentiation, whereas a direct effect on progenitor-cell proliferation has not been demonstrated. Its possible contributions to muscle regeneration, hypertrophy, immune communication, and systemic exercise adaptation remain insufficiently established.

- ***CXCL8 (IL-8)***

CXCL8, historically known as interleukin-8 (IL-8), is an ELR-positive CXC chemokine with potent chemotactic properties [24]. Skeletal muscle cells can express and secrete CXCL8, supporting its classification as a cytokine-type myokine [20,25]. In human skeletal muscle, contractile activity increases CXCL8 gene expression, and the magnitude of this response appears to be influenced by intramuscular glycogen availability [26]. Primary human myotubes also secrete CXCL8, and electrical pulse stimulation increases its transcription and release, providing direct evidence that contracting muscle cells can constitute a source of this chemokine [25]. Nevertheless, the extent to which skeletal muscle contributes to circulating CXCL8 during physiological exercise remains less clearly established than for IL-6 [20].

Muscular CXCL8 production is not restricted to physiological contraction. Its expression can also be induced by inflammatory cytokines, oxidative stress, metabolic disturbances, and tissue damage [27,28]. Moreover, endothelial cells, fibroblasts, macrophages, and other infiltrating leukocytes within skeletal muscle may also produce CXCL8; therefore, tissue-level expression alone does not establish a myofiber source [20,28,29,30].

CXCL8 signals primarily through the G-protein-coupled receptors CXCR1 and CXCR2 [24]. Both receptors are expressed by neutrophils, whereas exercise-induced CXCR2 appears to have a particularly important role in endothelial responses [24,31]. Receptor activation can engage several intracellular pathways, including MAPK/ERK and PI3K/Akt signaling [32,33]. The relative involvement of these pathways depends on the target cell, receptor expression, CXCL8 concentration, and duration of exposure.

The most firmly established local action of muscle-derived CXCL8 is the regulation of the microvasculature [24,31]. CXCL8 can promote endothelial-cell survival, proliferation, migration, and organization into capillary-like structures. It can also increase the production of matrix metalloproteinases, thereby facilitating extracellular-matrix remodeling and endothelial invasion during new-vessel formation. Through these predominantly paracrine effects, contraction-induced CXCL8 may contribute to the coordination of muscle activity and metabolic demand with capillary remodeling [24,31]. Nevertheless, its specific contribution to exercise-induced skeletal muscle angiogenesis in humans has not been established as conclusively as that of VEGF-A [20,31]. CXCL8 may also participate in the inflammatory response to muscle injury. It is a potent chemoattractant and activator of neutrophils, acting through CXCR1 and CXCR2 to promote their migration toward sites of tissue stress or damage [24]. Locally generated CXCL8 could therefore facilitate the early recruitment of neutrophils to injured muscle and contribute to the removal of damaged material and initiation of tissue remodeling [24,30]. However, neutrophil recruitment can be beneficial or detrimental depending on its magnitude and duration, and the specific contribution of myofiber-derived CXCL8, as opposed to CXCL8 produced by endothelial, stromal, or immune cells, remains insufficiently defined [20,29,30].

Direct effects of CXCL8 on muscle cells have also been proposed, although the available evidence remains limited and apparently context-dependent. In cultured rat skeletal muscle cells, CXCL8 enhanced Akt–FoxO3 signaling, promoted myogenic differentiation, and produced effects interpreted as anticatabolic [34]. These findings suggest that locally produced CXCL8 could support muscle-cell survival or differentiation under selected physiological conditions. However, these actions have not yet been adequately confirmed in human muscle or in vivo models of normal muscle adaptation.

By contrast, sustained or excessive CXCL8 signaling may have adverse muscular and vascular consequences. Primary myotubes obtained from individuals with type 2 diabetes secreted greater amounts of CXCL8 than myotubes from individuals without diabetes, and experimental evidence implicated this excessive production in impaired endothelial tube growth [32]. In another pathological context, CXCL8 released by pancreatic cancer cells and tumor-associated stromal cells induced myotube atrophy through a CXCR2–ERK1/2-dependent mechanism [33]. These apparently opposing observations indicate that the biological effects of CXCL8 cannot be classified as intrinsically angiogenic, regenerative, or catabolic, but depend on its source, concentration, receptor distribution, and duration of exposure.

Overall, muscle-derived CXCL8 can be regarded as a contraction-, stress-, and injury-responsive predominantly paracrine myokine with possible autocrine actions. Its best-supported local functions involve endothelial-cell regulation, proangiogenic signaling, extracellular-matrix remodeling, and possible leukocyte recruitment, whereas direct effects on muscle-cell differentiation and protein homeostasis remain less firmly established. Transient and spatially restricted CXCL8 production may contribute to vascular adaptation and the early response to muscle damage, whereas excessive or sustained signaling may impair vascular homeostasis or, in pathological settings, promote muscle catabolism.

- ***Interleukin 15 (IL-15)***

IL-15 is a pleiotropic cytokine structurally and functionally related to IL-2 and is widely regarded as a cytokine-type myokine [20,35,36]. Skeletal muscle is one of the tissues with relatively high IL-15 gene expression, and both muscle fibers and cultured myogenic cells can produce this cytokine or IL-15/IL-15Rα complexes, although translation, intracellular trafficking, membrane presentation, and secretion are tightly regulated [36,37]. Human skeletal muscle IL15 expression appears to vary according to fiber composition, with higher transcript abundance reported in muscles containing a greater proportion of type II fibers. Resistance exercise can transiently increase muscular IL15 mRNA, although this transcriptional response is not necessarily accompanied by a parallel increase in muscle or plasma IL-15 protein [36]. These observations indicate that IL-15 production is subject to substantial post-transcriptional and translational regulation.

The classification of IL-15 as an exercise-regulated endocrine myokine nevertheless requires some caution [20,24]. Acute exercise studies have reported variable changes in muscle IL15 expression and circulating IL-15 concentrations. In one human study, acute cycling did not immediately increase muscle IL15 expression and produced only a modest rise in plasma IL-15, whereas resistance-exercise studies have reported changes in muscular IL-15/IL-15Rα signaling associated with myofibrillar protein synthesis [38]. Because net muscular release has not been demonstrated, IL-15 should therefore be considered primarily a locally regulated, potentially cell-associated myokine with a possible, but less firmly established, endocrine role [38]. IL-15 signaling differs from that of many conventionally secreted cytokines. IL-15 binds with high affinity to the IL-15 receptor α-chain (IL-15Rα), and the resulting complex can be presented at the cell surface to adjacent cells expressing the signaling receptor subunits IL-2/IL-15Rβ, also known as CD122, and the common cytokine receptor γ-chain, CD132. This mechanism, termed trans-presentation, is central to IL-15 biology [39]. Activation of the receptor complex engages JAK1 and JAK3 and subsequently activates STAT3 and STAT5. Depending on the target cell, PI3K/Akt and MAPK-related pathways may also participate. IL-15Rα additionally contributes to the intracellular stability, transport, membrane presentation, and secretion of IL-15 [39].

One of the earliest proposed actions of IL-15 in skeletal muscle was the stimulation of muscle-fiber growth. In cultured differentiated myotubes, IL-15 increased protein accumulation and myotube size without clearly stimulating myoblast proliferation, suggesting a predominantly hypertrophic rather than mitogenic effect [35,40]. However, these effects are not uniform across experimental systems and may depend on the concentration and molecular form of IL-15, the presence of IL-15Rα, the differentiation state of the target cell, and the duration of exposure [24,35,41]. More recent evidence indicates that IL-15 may also regulate human myogenesis. In primary human muscle cultures, IL-15 enhanced myogenic differentiation and myotube development and partially counteracted the detrimental effects of TNF-α [42]. These findings differ somewhat from earlier observations in murine myotubes, in which hypertrophy occurred without marked changes in proliferation or differentiation. IL-15 may therefore exert stage- and model-dependent actions, supporting differentiation under inflammatory or regeneration-related conditions while acting predominantly on protein accumulation in mature myotubes. The extent to which these effects contribute to normal human muscle adaptation in vivo remains uncertain [42]. IL-15 also participates in skeletal muscle regeneration. Experimental studies indicate that IL-15 promotes recovery following muscle injury partly by regulating fibro-adipogenic progenitors, a stromal-cell population that supports satellite-cell-mediated regeneration and larger regenerating myofibers but can contribute to fibrosis and fatty infiltration when inadequately controlled [43]. Loss or inhibition of IL-15 signaling impairs the regenerative response, whereas increased IL-15 activity favors restoration of muscle structure and limits inappropriate fibro-adipogenic expansion. Another study found normal cardiotoxin-induced regeneration and compensatory hypertrophy in mice lacking skeletal muscle-derived IL-15 [37]. Together, these apparently divergent findings suggest that IL-15 can modulate the regenerative environment but is not universally required for muscle regeneration or compensatory hypertrophy [37,43]. This mechanism has been demonstrated mainly in animal models and requires further confirmation in human muscle injury. Metabolic actions have also been attributed to muscle-derived IL-15 [24,35]. In experimental muscle systems, IL-15 increases glucose uptake, activates AMP-activated protein kinase, and enhances mitochondrial oxidative metabolism. These effects have been associated with increased formation of mitochondrial respiratory-chain supercomplexes and improved capacity for fatty-acid oxidation [44]. Transgenic overexpression of IL-15 in mice also produces a more oxidative muscle phenotype, increases endurance capacity, and favors lipid utilization during exercise. Collectively, these findings suggest that IL-15 may contribute to metabolic flexibility and the adaptation of skeletal muscle to sustained energy demand. However, most mechanistic evidence derives from cultured cells and genetically modified animals rather than physiological concentrations of IL-15 in humans [44]. Muscle-derived IL-15 may additionally mediate communication between skeletal muscle and adipose tissue [35,45]. Mice engineered to oversecrete IL-15 from skeletal muscle exhibit elevated circulating IL-15 and reduced adipose-tissue mass, including partial protection against diet-induced adiposity. Proposed mechanisms include increased lipid oxidation, enhanced adipose-tissue energy expenditure, and modulation of adipocyte metabolism. Nevertheless, these models produce sustained IL-15 concentrations that may exceed those normally observed following exercise. Evidence that physiological secretion of IL-15 from human muscle substantially controls adiposity or whole-body energy expenditure remains limited. IL-15 also constitutes a potential link between skeletal muscle and the immune system. It is a major survival and activation factor for natural killer cells and memory CD8-positive T cells. Locally produced muscular IL-15 could therefore influence immune surveillance and immune-cell behavior within the muscle microenvironment. In experimental inflammatory myopathy, skeletal muscle-derived IL-15 enhanced CD8-positive T-cell function, indicating that local IL-15 signaling may contribute to persistent immune-mediated muscle injury [39]. Thus, although transient IL-15 signaling may support tissue adaptation and regeneration, sustained or dysregulated production could promote local cytotoxic immune responses under pathological conditions. IL-15 has also been investigated as a protective factor against muscle wasting. Experimental administration or overexpression of IL-15 reduces muscle protein degradation and attenuates loss of muscle mass in some models of cancer cachexia [46]. In human myogenic cultures, IL-15 can partly protect myotube development from TNF-α-mediated impairment [42]. However, clinical evidence remains insufficient to establish that reduced muscle-derived IL-15 causes sarcopenia or cachexia, or that increasing IL-15 would safely restore muscle mass. Its potent immunostimulatory activity and the possibility of promoting chronic inflammatory responses must be considered when evaluating its therapeutic potential.

Overall, muscle-derived IL-15 can be regarded as a predominantly local and potentially endocrine myokine involved in the regulation of muscle growth, myogenic differentiation, the regenerative environment, oxidative metabolism, and communication with adipose and immune tissues. Its best-supported local actions include promotion of myotube development, attenuation of muscle protein loss in selected experimental models, metabolic remodeling, and regulation of the cellular environment during muscle repair. Its proposed systemic effects on adiposity and energy expenditure are supported mainly by animal overexpression models and remain less clearly demonstrated under physiological conditions in humans, whereas sustained or dysregulated IL-15 signaling may enhance cytotoxic immune responses and inflammatory muscle damage.

- ***CCL2 (MCP-1)***

CCL2, historically known also as monocyte chemoattractant protein-1 (MCP-1), is a member of the CC chemokine family and a potent regulator of monocyte and macrophage trafficking. Skeletal muscle cells can express and secrete CCL2, supporting its classification as a cytokine-type myokine [20,28,47]. In cultured myotubes, electrical stimulation increases CCL2 expression and release through mechanisms involving intracellular calcium and nuclear factor κB (NF-κB) signaling. In human skeletal muscle, an acute bout of resistance exercise similarly increased NF-κB binding to the CCL2 promoter and induced muscular CCL2 mRNA expression [48]. These findings indicate that contractile activity can directly activate CCL2 production within muscle cells. However, net release of CCL2 from exercising human muscle has not been directly established [20].

Muscular CCL2 production is particularly prominent after tissue injury. In experimental models of acute muscle damage, CCL2 is expressed by injured or regenerating myofibers as well as by mononuclear, endothelial, fibro-adipogenic, and other stromal or inflammatory cell populations [30,49]. Thus, the relative myogenic contribution to local CCL2 production varies according to the cellular composition of the injured muscle environment [20,47]. CCL2 signals predominantly through C-C chemokine receptor type 2 (CCR2), a G-protein-coupled receptor highly expressed by circulating inflammatory monocytes and their macrophage descendants [49]. CCR2 is also expressed by some myogenic progenitor cells, indicating that CCL2 may act not only indirectly through immune-cell recruitment but also directly on muscle-cell populations [50]. Depending on the target cell, CCR2 activation engages pathways involved in intracellular calcium mobilization, cytoskeletal remodeling, migration, proliferation, and cell survival.

The best-established action of locally produced CCL2 is the recruitment of CCR2-positive monocytes from the bone marrow and circulation into damaged skeletal muscle. Genetic deletion of CCL2 markedly reduces monocyte mobilization and macrophage accumulation after acute muscle injury. This impaired recruitment is accompanied by defective removal of necrotic material, reduced macrophage expression of insulin-like growth factor 1, and delayed muscle-fiber regeneration. Local administration of IGF-1 partially restores regeneration in CCL2-deficient muscle, indicating that CCL2 supports repair partly by recruiting macrophages that subsequently provide trophic and regenerative signals [49]. The functional importance of this pathway is also demonstrated by studies targeting CCR2. Mice lacking CCR2 show reduced early macrophage accumulation, impaired regeneration, increased fatty or fibrotic replacement, and delayed recovery of muscle force after injury [50]. Neutralization of CCL2 similarly compromises functional recovery [51]. Thus, the CCL2-CCR2 axis does not merely produce an inflammatory-cell infiltrate; it organizes a necessary early phase of tissue clearance and prepares the local environment for successful regeneration. Nevertheless, the effects of recruited macrophages depend on their subsequent phenotypic and functional transition. Persistent accumulation of inflammatory macrophages without timely progression toward reparative states may delay rather than facilitate tissue recovery [30]. CCL2 may additionally exert direct effects on myogenic cells. Experimental studies show that myoblasts express functional CC chemokine receptors and respond to CCL2 with changes in migration and proliferation [52]. This direct chemotactic activity could help mobilize satellite-cell-derived progenitors from adjacent regions toward areas of muscle damage. More recent experimental evidence also suggests that CCL2 can support myogenic differentiation and recovery of muscle performance [53]. However, these direct effects have been studied mainly in cultured cells and animal models, and their quantitative importance relative to macrophage-mediated mechanisms remains uncertain. Contraction-induced CCL2 may also participate in muscle remodeling after strenuous or unfamiliar exercise. Resistance exercise increases CCL2 transcription in human skeletal muscle [48], and CCL2–CCR2 genetic variants have been associated with interindividual differences in muscle damage and recovery following eccentric exercise [54]. These observations are compatible with a role for CCL2 in coordinating immune-cell recruitment and tissue remodeling after exercise-induced stress. Nevertheless, genetic associations and post-exercise expression changes do not demonstrate that CCL2 is essential for normal training adaptation or hypertrophy in humans. Its role is likely to be more relevant when exercise produces substantial structural stress or damage than during non-damaging contractile activity. The relationship between CCL2 and muscle metabolism is less clearly defined. Chronic low-grade inflammation and increased CCL2 concentrations have been associated with obesity and insulin resistance, raising the possibility that persistent muscle-derived CCL2 impairs insulin action by recruiting inflammatory macrophages [45,55]. However, muscle-specific overexpression of CCL2 in mice produced marked macrophage infiltration and an inflammatory transcriptional profile without impairing skeletal muscle insulin signaling, glucose tolerance, or whole-body insulin sensitivity [55]. These findings indicate that local CCL2-driven inflammation is not, by itself, sufficient to cause metabolic dysfunction and that additional metabolic or inflammatory factors are probably required.

Sustained or inappropriate activation of the CCL2–CCR2 axis may nevertheless become detrimental in selected pathological settings. Persistent CCL2 production can maintain monocyte recruitment, prolong inflammatory-cell accumulation, and contribute to fibrosis, fatty replacement, or disruption of the neuromuscular environment [30,50]. Recent experimental evidence in amyotrophic lateral sclerosis models indicates that local CCL2–CCR2 signaling promotes immune-cell infiltration around neuromuscular junctions and contributes to denervation [56]. These pathological effects differ from the transient, spatially restricted CCL2 response required for efficient repair after acute injury [49].

Overall, muscle-derived CCL2 can be regarded as a contraction-, injury-, and stress-responsive predominantly paracrine myokine whose best-established function is the coordination of monocyte and macrophage recruitment through CCR2. By promoting early tissue clearance and macrophage-derived trophic signaling, transient CCL2 activity supports muscle regeneration and functional recovery, whereas its direct effects on myogenic cells and its contribution to physiological training adaptation remain less firmly established. Sustained CCL2–CCR2 activation may instead perpetuate inflammation and contribute to pathological remodeling or neuromuscular damage.

- ***CXCL1 (GROα)***

CXCL1, historically known as growth-regulated oncogene α (GROα), is an ELR-positive member of the CXC chemokine family. The murine ortholog is commonly referred to as CXCL1/KC. Skeletal muscle cells can produce and secrete CXCL1, supporting its classification as a cytokine-type myokine. Electrical pulse stimulation of contractile mouse myotubes rapidly increased CXCL1 expression and release, and treadmill exercise increased its expression in mouse skeletal muscle. Primary human myotubes similarly secreted CXCL1, with electrical stimulation further increasing its production. These findings demonstrate that both murine and human muscle cells can act as sources of CXCL1 in response to contractile activity [20,28,57,58,59]. Contraction-induced CXCL1 production appears to occur relatively rapidly. In cultured mouse myotubes, CXCL1 was induced within the first hours of electrical stimulation, earlier than the more delayed IL-6 response. In cultured mouse myotubes, this rapid induction depended on JNK- and NF-κB-dependent pathways, whereas ERK1/2, p38, and calcineurin were not required. Mechanical stretch can also stimulate CXCL1 expression, indicating that both active contraction and mechanical deformation may contribute to its production [57]. In mice, exercise markedly increases CXCL1 expression in the liver as well as in skeletal muscle, with the hepatic response being particularly prominent and partly regulated by muscle-derived IL-6. Thus, skeletal muscle may influence circulating CXCL1 both through its own production and indirectly by regulating production in distant organs. Direct evidence of substantial net CXCL1 release from exercising human muscle remains limited, and its endocrine role in normal human exercise is therefore less firmly established than that of IL-6 [2,20,58].

CXCL1 signals predominantly through C-X-C chemokine receptor type 2 (CXCR2), a G-protein-coupled receptor expressed by neutrophils and several non-immune cell populations. In murine myogenic cells, CXCL1–CXCR2 signaling activates pertussis-toxin-sensitive Gαi proteins and increases intracellular calcium concentrations. Depending on the target cell, CXCR2 activation can engage additional signaling pathways controlling cytoskeletal remodeling, migration, proliferation, and cell survival. CXCR2 expression and the presence of other receptor ligands are therefore important determinants of the biological response to CXCL1 [31,57].

One of the proposed local actions of muscle-derived CXCL1 is the regulation of myogenic progenitor cells. In C2C12 myoblasts, both CXCL1 and the related chemokine CXCL5 increased intracellular calcium and stimulated cell migration. CXCR2-mediated signaling also contributed to myogenic differentiation in this experimental model. These findings suggest that contraction-induced CXCL1 may facilitate the movement of myogenic progenitors toward regions undergoing adaptation or repair and influence their progression through the myogenic program. However, these direct actions have been demonstrated principally in murine cell lines and require confirmation in primary human cells and in vivo muscle regeneration [57]. CXCL1 also acts as a potent neutrophil chemoattractant. In skeletal muscle, a potentially protective example of this recruitment has been described following denervation. In a mouse model of denervation, muscular CXCL1 expression increased rapidly and recruited a transient population of CXCR2-positive neutrophils. Experimental blockade of neutrophil recruitment or activity accelerated early muscle atrophy, suggesting that their temporary recruitment exerted a protective effect during the first phase following denervation. The consequences of neutrophil recruitment are nevertheless context-dependent and cannot be inferred from this denervation model alone. The denervation model demonstrated that the recruited neutrophils progressively disappeared through a p53-associated apoptotic mechanism. This identifies a mechanism controlling their persistence but does not demonstrate that apoptosis was required for their protective effect; indeed, p53 deficiency reduced neutrophil apoptosis and was associated with less severe early atrophy [60]. Metabolic functions have also been attributed to muscle-derived CXCL1. In mice subjected to electrotransfer-mediated CXCL1 overexpression in skeletal muscle, increased circulating CXCL1 was associated with greater muscular fatty-acid oxidation and oxidative capacity, reduced diet-induced adiposity, and improved metabolic phenotype. These results suggest that CXCL1 may promote muscular lipid utilization and indirectly influence adipose accumulation. However, sustained overexpression does not reproduce the magnitude or temporal pattern of physiological exercise-induced secretion, and the metabolic significance of endogenous muscle-derived CXCL1 in humans remains uncertain [2,45,61].

CXCL1 may also participate in communication between skeletal muscle and tumors. Electrical pulse stimulation of human myotubes increases the release of CXCL1 together with other cytokines, and conditioned media containing these factors reduced the viability or growth of selected pancreatic cancer-cell models. Nevertheless, these antitumor effects cannot be attributed exclusively to CXCL1, because the muscle secretome contains multiple interacting mediators. Recombinant CXCL1 also affected selected pancreatic cancer-cell lines, but these findings should not be generalized to other tumor microenvironments [20,59].

Overall, muscle-derived CXCL1 can be regarded as a contraction-, mechanical-stress-, and injury-responsive myokine with predominantly local autocrine or paracrine actions, and a less firmly established endocrine role. Its best-supported functions include CXCR2-dependent signaling in myogenic cells and recruitment of neutrophils to stressed or damaged muscle, whereas its proposed metabolic effects on fatty-acid oxidation and whole-body adiposity are supported mainly by animal models and remain uncertain in humans. Transient and spatially restricted CXCL1 signaling may support muscle adaptation, controlled inflammatory responses, and repair, whereas excessive or prolonged signaling may perpetuate inflammation and collateral tissue damage.

- ***CXCL10 (IP-10)***

CXCL10, historically known as interferon-γ-induced protein 10 (IP-10), is a member of the non-ELR CXC chemokine family. Skeletal muscle cells can produce and secrete CXCL10, supporting its classification as a cytokine-type myokine. However, CXCL10 differs from most classical exercise-induced myokines because contractile activity appears to reduce, rather than stimulate, its secretion in some experimental models. It may therefore be more appropriately described as an inflammation-, metabolic-stress-, and damage-responsive myokine whose production is modulated by exercise [20,28,45,62]. In cultured C2C12 myotubes, CXCL10 was detectable in the conditioned medium under basal conditions, whereas electrical pulse stimulation significantly reduced both its expression and secretion. Conditioned medium from contracting myotubes increased endothelial-cell viability compared with medium from non-contracting cells, and addition of recombinant CXCL10 attenuated this effect. These findings led to the proposal that contraction-dependent suppression of muscle-derived CXCL10 may facilitate vascular adaptation by removing an inhibitory signal rather than by releasing a conventional pro-angiogenic myokine. Nevertheless, this mechanism has been demonstrated mainly in murine cultured cells and has not been confirmed through measurements of CXCL10 release from exercising human muscle [62]. Muscular CXCL10 production is strongly stimulated by inflammatory signals. Human skeletal muscle cells exposed to interferon-γ or tumor necrosis factor α secreted CXCL10, with a marked synergistic response when both cytokines were combined. Interferon-γ-induced production involved STAT1 signaling, whereas the response to tumor necrosis factor α involved NF-κB activation. This ability of muscle cells to amplify local chemokine production suggests that they can actively participate in inflammatory processes rather than functioning solely as passive targets of immune-mediated damage [45,63]. CXCL10 signals principally through C-X-C chemokine receptor type 3 (CXCR3), which is prominently expressed by activated and memory T lymphocytes and by selected natural killer and other immune-cell populations. Activation of CXCR3 promotes cytoskeletal reorganization, adhesion, chemotaxis, and migration toward areas containing a CXCL10 gradient. CXCL9 and CXCL11 also bind CXCR3, creating substantial functional overlap among these chemokines. Consequently, inhibition or deletion of CXCL10 may be partially compensated for by increased signaling through the other CXCR3 ligands [64].

A major proposed local action of muscle-derived CXCL10 is the recruitment and retention of activated T cells within an inflammatory muscle environment. In inflammatory myopathies, interferon-γ- and tumor necrosis factor α-induced CXCL10 production by muscle cells may attract CXCR3-positive Th1 and cytotoxic T cells, which can subsequently release additional inflammatory mediators and stimulate further CXCL10 production. This may create a feed-forward circuit between muscle cells and infiltrating lymphocytes that may perpetuate tissue inflammation. In this context, CXCL10 is better regarded as a pathological inflammatory myokine than as a mediator of normal exercise adaptation [45,63]. CXCL10 also increases following contraction-induced muscle damage. In human skeletal muscle, its abundance was elevated 24 and 72 h after damaging exercise. In primary human myoblast cultures, recombinant CXCL10 did not alter cell proliferation but promoted myogenic differentiation, suggesting a possible direct effect on the progression of myogenic progenitors. However, CXCL10-deficient mice showed no clear impairment in muscle-force recovery, formation of regenerating fibers, or T-cell accumulation following experimental injury. Increased expression of both CXCL9 and CXCL11 may have compensated for the absence of CXCL10. Thus, CXCL10 can influence myogenic differentiation experimentally but does not appear to be individually essential for effective muscle regeneration [64]. CXCL10 additionally has anti-angiogenic properties. Consistent with this property, recombinant CXCL10 attenuated the increase in endothelial-cell viability induced by conditioned medium from contracting C2C12 myotubes. In skeletal muscle, a reduction in CXCL10 secretion during contraction could therefore contribute to exercise-induced angiogenesis by relieving this anti-angiogenic restraint. This mechanism would complement the increased production of pro-angiogenic myokines such as VEGF-A and CXCL8. However, the specific contribution of muscle-derived CXCL10 suppression to capillary adaptation in humans remains unproven [20,28,62]. Potential endocrine effects of muscle-derived CXCL10 have also been proposed. Conditioned medium from electrically stimulated mouse myotubes increased collagen production by dermal fibroblasts, and pharmacological inhibition of CXCR3 similarly increased collagen production in fibroblasts exposed to conditioned medium from non-contracting myotubes. Conversely, addition of recombinant CXCL10 attenuated the contraction-conditioned-medium-induced increase in collagen production. These findings suggest that contraction-associated suppression of muscular CXCL10 might influence connective-tissue remodeling in distant tissues. However, this proposed muscle–skin communication pathway has so far been demonstrated only in murine cell-culture systems [65]. Metabolic conditions may also modify muscular CXCL10 production. Elevated glucose and palmitate concentrations increased CXCL10 expression in cultured myotubes, while high-fat feeding increased its expression in mouse skeletal muscle. Given the anti-angiogenic and immune-cell-recruiting properties of CXCL10, persistent muscular production during metabolic disease could potentially contribute to impaired microvascular adaptation or chronic local inflammation. Nevertheless, direct evidence that muscle-derived CXCL10 causes metabolic dysfunction or capillary rarefaction in humans remains insufficient [45].

Overall, muscle-derived CXCL10 can be regarded as an inflammation-, metabolic-stress-, and damage-responsive predominantly paracrine myokine with possible direct actions on myogenic cells, whose secretion may be suppressed by contractile activity. Its best-established functions involve CXCR3-dependent immune-cell recruitment, amplification of Th1-type inflammation within muscle, and inhibition of angiogenic responses, whereas its effects on myogenic differentiation and connective-tissue signaling remain less firmly established and its proposed endocrine actions remain preliminary. Transient CXCL10 expression following muscle damage may participate in immune coordination and tissue remodeling, whereas sustained production may perpetuate inflammation and restrain vascular adaptation.

- ***Irisin***

Irisin is a peptide myokine generated through proteolytic processing of fibronectin type III domain-containing protein 5 (FNDC5), a type I transmembrane glycoprotein expressed in skeletal muscle and several other tissues. FNDC5 was initially identified as a downstream target of peroxisome proliferator-activated receptor γ coactivator 1α (PGC-1α) in exercising skeletal muscle. Cleavage of its extracellular domain was proposed to release irisin, a 112-amino-acid peptide capable of acting through autocrine, paracrine, and endocrine mechanisms. The original identification of irisin linked muscular contractile activity to the browning of white adipose tissue and increased systemic energy expenditure in mice [20,66]. FNDC5 expression and irisin production are regulated during myogenesis. In primary human muscle cells, FNDC5 expression and irisin secretion increased as myoblasts differentiated into myotubes, in parallel with increases in PGC-1α and myogenin. These observations indicate that differentiated muscle cells can produce irisin and support its classification as a genuine muscle-derived factor [67]. Skeletal muscle is not, however, its exclusive source, as FNDC5 is also expressed in adipose tissue, the heart, brain, and other organs. Consequently, circulating irisin cannot automatically be attributed to skeletal muscle [20,67,68]. The regulation of muscle-derived irisin by exercise in humans remains less consistent than initially proposed. Some studies have reported increases in muscular FNDC5 expression or circulating irisin after acute exercise or training, whereas others have found little or no response. Targeted tandem mass spectrometry has confirmed that irisin is present in human plasma and reported a modest increase following aerobic interval training in a small cohort. However, immunoassay-based studies have produced circulating concentrations differing by several orders of magnitude, raising concerns regarding antibody specificity and the comparability of published measurements. Irisin can therefore be considered an established circulating human protein, but the magnitude and consistency of its exercise-induced release remain uncertain [20,69,70]. αV integrins, particularly αVβ5, are currently the best-supported receptors for irisin. Direct interaction between irisin and αVβ5 has recently been demonstrated in C2C12 myoblasts, with activation of FAK/AKT/mTOR-related signaling. Whether the same receptor complex mediates irisin actions in mature human muscle fibers remains to be demonstrated [71]. Experimental exposure of muscle cells to recombinant irisin activates several intracellular pathways, including ERK1/2, PI3K/Akt/mTOR, and AMP-activated protein kinase. In myogenic cultures, irisin can also induce IL-6-related signaling. Nevertheless, the receptor and pathway involved may differ according to the target cell, concentration of irisin, glycosylation state, and experimental preparation [71,72,73].

One of the best-supported actions of irisin within skeletal muscle is the regulation of myogenesis. In cultured human myocytes, recombinant irisin increased the expression of insulin-like growth factor 1 and the hypertrophy-associated PGC-1α4 isoform while reducing myostatin expression through an ERK-dependent mechanism [67]. In murine and primary human myogenic cultures, irisin also enhanced myoblast differentiation, myotube formation, and cell fusion. These pro-myogenic effects were associated with increased expression of the fusion gene myomaker, whereas caveolin-3 showed only a non-significant upward trend. They were also partly dependent on induction of IL-6 signaling [72]. Animal studies further suggest that irisin promotes muscle hypertrophy and regeneration. Administration of recombinant irisin to uninjured mice increased muscle-fiber size and grip strength. Following experimental muscle injury, irisin enhanced satellite-cell activation, increased the number of proliferating MyoD-positive myogenic cells, activated signaling pathways consistent with increased protein synthesis and improved the formation of regenerating fibers. Irisin also partially protected against denervation-induced muscle loss by stimulating satellite-cell activity and reducing the expression of the ubiquitin ligases atrogin-1 and MuRF1 [72]. These findings support a pro-myogenic and potentially anticatabolic action, although they have not yet been confirmed through comparable intervention studies in humans. Recent evidence using FNDC5 overexpression nevertheless suggests that αVβ5 activation can favor myoblast proliferation over differentiation in some experimental settings, indicating that the myogenic outcome may depend on the mode and duration of FNDC5/irisin exposure and on the cellular signaling context [71]. Irisin may also regulate muscle glucose metabolism. In cultured skeletal muscle cells, recombinant irisin increased glucose uptake and GLUT4 translocation through a mechanism involving transient reactive oxygen species generation, AMPK activation, and downstream signaling [73]. These effects suggest that locally produced irisin could contribute to the adjustment of glucose utilization during increased energy demand. However, the concentrations used in cellular experiments may exceed physiological exposure, and the contribution of endogenous muscle-derived irisin to glucose disposal during human exercise remains uncertain. The endocrine actions of irisin were initially characterized in adipose tissue. Experimental increases in FNDC5 or irisin promoted the expression of uncoupling protein 1 and other thermogenic genes in white adipocytes, producing a brown- or beige-like phenotype and improving glucose homeostasis in mice [66]. The extent to which physiological irisin concentrations induce comparable adipose-tissue browning in humans remains debated, as some studies using human adipocytes have failed to reproduce the effects observed in murine models [67,70,74]. These systemic actions should therefore be distinguished from the more direct pro-myogenic effects described in muscle-cell and animal models.

Several methodological considerations complicate the interpretation of irisin research. Recombinant irisin preparations differ in source, purity, glycosylation, and biological activity, and many experimental studies have used concentrations substantially higher than those detected in human plasma by mass spectrometry. In addition, circulating irisin measurements obtained using different immunoassays are frequently inconsistent. Associations between serum irisin and muscle mass, exercise capacity, metabolic disease, or sarcopenia should therefore not be interpreted automatically as evidence of causation or of muscular origin [20,69,70,72,73].

Overall, muscle-derived irisin can be regarded as a differentiation- and potentially contraction-regulated myokine with local autocrine and paracrine actions, and a possible endocrine role. Its best-supported actions in experimental muscle models include stimulation of myogenic differentiation and fusion, activation of satellite cells, activation of anabolic signaling and promotion of hypertrophy, support of regeneration, and regulation of glucose uptake. Nevertheless, most causal evidence for these effects derives from cultured cells and animal models, while their physiological relevance in human muscle remains less firmly established. The biological consequences of irisin are likely to depend on its tissue source, concentration, molecular processing and glycosylation, receptor availability, duration of exposure, and physiological or pathological context.

- ***Myostatin (GDF-8)***

Myostatin, also known as growth differentiation factor 8 (GDF-8), is a secreted member of the transforming growth factor β superfamily and one of the best-established muscle-derived growth regulators. It is expressed predominantly in skeletal muscle, where it is produced by developing and mature muscle fibers and acts mainly through autocrine and paracrine mechanisms. Myostatin is also detectable in the circulation, although much of it is present in latent or inhibitor-bound complexes; circulating abundance therefore does not directly indicate systemic biological activity. In contrast to most myokines and growth factors discussed above, myostatin functions primarily as a negative regulator that limits skeletal muscle development, postnatal fiber growth, and regenerative activity. Genetic deletion of *MSTN* in mice produces a marked increase in skeletal muscle mass through a combination of muscle-fiber hyperplasia and hypertrophy, providing the original functional evidence for its role as a muscle-growth suppressor [75,76].

Myostatin is synthesized as a precursor protein containing an amino-terminal propeptide and a carboxy-terminal mature signaling domain. Following proteolytic processing, the mature carboxy-terminal fragments form a disulfide-linked dimer that remains non-covalently associated with the propeptide in a latent complex. Additional cleavage of the propeptide by members of the BMP-1/tolloid family of metalloproteinases releases biologically active myostatin [75,77]. Its availability is further controlled extracellularly by endogenous binding proteins, including follistatin, FSTL3, the myostatin propeptide, and GASP-family proteins. Myostatin activity therefore depends not only on its expression but also on precursor processing, extracellular activation, and the local balance between the mature ligand and its inhibitors [41,75,77]. Muscular myostatin expression is regulated by developmental, mechanical, metabolic, and pathological signals. Disuse and unloading can increase its expression, whereas acute and chronic exercise have frequently been associated with reduced *MSTN* mRNA in human skeletal muscle. Nevertheless, the response varies according to exercise modality, training status, sampling time, and the molecular form measured. Changes in muscular gene expression do not necessarily correspond to changes in active protein secretion or circulating myostatin. Exercise-related reductions in *MSTN* expression should therefore be interpreted as evidence of altered local growth regulation rather than direct proof that systemic myostatin activity has decreased [20,28]. Myostatin initially binds to the activin type II receptors ACVR2B and ACVR2A, with ACVR2B generally displaying greater affinity. The ligand–receptor complex subsequently recruits the type I receptors ALK4 or ALK5. Because these receptor components are shared with other transforming growth factor β-superfamily ligands, interventions acting at the receptor level are not necessarily specific for myostatin. Receptor activation leads predominantly to phosphorylation of SMAD2 and SMAD3, which associate with SMAD4 and translocate to the nucleus to regulate transcription. Myostatin signaling also interacts with MAPK, PI3K/Akt, mTOR, and other pathways controlling cell proliferation, differentiation, protein synthesis, and muscle-fiber size [41,75].

The best-established action of myostatin is the limitation of skeletal muscle mass. Its physiological importance has been demonstrated across species. Naturally occurring *MSTN* mutations produce the double-muscling phenotype in several cattle breeds, while a function-disrupting mutation identified in a child was associated with pronounced muscle hypertrophy and increased strength. These observations confirm that myostatin is an active regulator of human as well as animal muscle development [75,76,78]. However, increased muscle size following myostatin deficiency or inhibition does not necessarily produce an equivalent increase in specific force, endurance, or overall physical function, because muscle architecture, connective tissue, innervation, and metabolic phenotype may also be altered [41,75]. Myostatin regulates muscle growth partly through direct actions on myogenic progenitor cells. In cultured myoblasts, it inhibits cell-cycle progression and reduces proliferation through mechanisms involving the regulation of cyclins and cyclin-dependent kinase inhibitors. Myostatin also suppresses the expression and activity of myogenic regulatory factors and inhibits myoblast differentiation and fusion into multinucleated myotubes. These effects restrict both the number of progenitor cells available for growth and their progression toward terminal differentiation [41,79]. Satellite cells also appear to be regulated by myostatin. Experimental studies indicate that myostatin helps maintain satellite-cell quiescence and restrains their activation and proliferation. Conversely, genetic absence or pharmacological antagonism of myostatin has been associated in some experimental models with increased satellite-cell activation and the expression of myogenic markers after injury. Myostatin inhibition has accelerated regeneration and increased the size of newly formed fibers in several animal models. Nevertheless, satellite-cell responses are influenced by the nature and severity of the injury, the timing of myostatin inhibition, and interactions with inflammatory and stromal cells. Myostatin should therefore be regarded as an important brake on regenerative activation rather than as an absolute inhibitor of every phase of muscle repair [41,79]. In mature muscle fibers, myostatin opposes anabolic signaling. Experimental exposure to myostatin reduces Akt activation and suppresses mTOR complex 1 and p70 S6 kinase signaling, thereby limiting pathways involved in protein synthesis, myotube differentiation, and fiber hypertrophy. Myostatin also reduces the diameter of differentiated myotubes [41,80]. These observations indicate that myostatin can constrain muscle-fiber size independently of its effects on progenitor-cell proliferation. The relationship between myostatin and protein degradation is more complex. Some experimental studies have associated excessive myostatin signaling with FoxO activation, ubiquitin–proteasome activity, autophagy, and increased expression of atrophy-related genes. However, other studies found that myostatin reduced myotube size primarily by suppressing differentiation-associated and anabolic programs without directly inducing the canonical atrogin-1/MAFbx and MuRF1 atrophy program. Thus, although myostatin can contribute to muscle wasting in selected pathological settings, its most consistently demonstrated direct effect is inhibition of anabolic and myogenic signaling rather than universal activation of proteolysis [41,80]. Myostatin also influences the non-myogenic components of skeletal muscle. It stimulates the proliferation of muscle-derived fibroblasts and increases the production of extracellular-matrix proteins, including collagen. Excessive myostatin activity may therefore contribute simultaneously to muscle-fiber atrophy and interstitial fibrosis, particularly during chronic injury, muscular dystrophy, aging, or other conditions characterized by defective regeneration. Conversely, myostatin inhibition can reduce fibrosis in some experimental models. These actions indicate that myostatin regulates not only muscle-cell size but also the structural environment in which regeneration and force transmission occur [41,81]. The role of myostatin in muscle metabolism is less clearly defined than its control of muscle mass. Myostatin deficiency and inhibition have been associated in animal models with reduced adiposity, improved insulin sensitivity, and changes in oxidative and glycolytic metabolism. However, many of these systemic effects may be secondary to the substantial increase in muscle mass or to the inhibition of additional ligands that share activin receptors. Consequently, results obtained using soluble ACVR2B receptors, follistatin, or other broad pathway inhibitors cannot be attributed exclusively to myostatin [41,75,82].

In pathological conditions, increased myostatin expression or signaling has been associated with disuse, denervation, glucocorticoid exposure, chronic systemic disease, cachexia, sarcopenia, and several neuromuscular disorders. Nevertheless, the direction and magnitude of these changes are not uniform, and circulating concentrations do not necessarily reflect biologically active myostatin within skeletal muscle. Moreover, muscle wasting is generally produced by multiple interacting pathways, and increased myostatin should not automatically be interpreted as its primary cause. The biological relevance of myostatin in each disease therefore requires evidence of local pathway activation, receptor engagement, and downstream signaling rather than measurement of total circulating protein alone [20,28,41,75].

Overall, muscle-derived myostatin can be regarded as a predominantly autocrine and paracrine myokine that acts as a physiological restraint on skeletal muscle growth and regenerative activity. Its best-established actions include inhibition of myoblast proliferation, restraint of satellite-cell activation, suppression of myogenic differentiation and fusion, attenuation of Akt–mTOR-dependent anabolic signaling, limitation of muscle-fiber hypertrophy, and promotion of fibroblast activity and extracellular-matrix deposition. Transient and appropriately regulated myostatin signaling may help maintain muscle-size homeostasis and restrain excessive progenitor-cell activation and expansion, whereas excessive or sustained activity may contribute to impaired regeneration, fibrosis, and muscle wasting. Its biological consequences therefore depend on the amount of active ligand, extracellular processing and inhibition, receptor availability, duration of signaling, and physiological or pathological context.

- ***Decorin***

Decorin is a small leucine-rich proteoglycan of the extracellular matrix that can also function as a muscle-derived signaling factor. Skeletal muscle cells express and release decorin, and its secretion increases in response to contraction. Circulating decorin concentrations also rise transiently after acute resistance exercise, supporting its classification as an exercise-regulated myokine, although skeletal muscle is not its exclusive tissue source [20,83].

The best-characterized action of decorin in skeletal muscle is its interaction with myostatin. Decorin can bind mature myostatin in a Zn^2+^-dependent manner and reduce its inhibitory activity on myogenic cells, supporting the proposal that extracellular-matrix-associated decorin may limit the local availability of active myostatin [84]. Experimental decorin overexpression is consequently associated with enhanced myogenic signaling and the expression of factors involved in muscle growth and differentiation [83]. Decorin may therefore contribute to exercise-induced hypertrophy by shifting the local balance away from myostatin-mediated growth inhibition [83,84].

Overall, muscle-derived decorin can be regarded as a contraction-responsive autocrine/paracrine myokine that may link extracellular-matrix remodeling with the regulation of muscle growth. Its best-supported function is the attenuation of myostatin activity, whereas its possible direct effects on myogenic-cell proliferation, differentiation, and regeneration remain less firmly established. Its biological consequences are likely to depend on its local concentration, extracellular-matrix interactions, timing of release, and the balance between decorin and active myostatin.

- ***Apelin***

Apelin is a bioactive peptide and an endogenous ligand of the G-protein-coupled apelin receptor, APLNR, formerly known as APJ. Skeletal muscle cells express and secrete apelin, and endurance training increases its muscular expression in humans. More specifically, increased skeletal-muscle apelin expression was demonstrated following endurance training in obese men. Muscle contraction also stimulates apelin production, supporting its classification as an exercise-regulated autocrine, paracrine, and potentially endocrine myokine [20,41,85,86,87]. However, skeletal muscle is not its only source, as apelin is also produced by adipose, cardiovascular, and other tissues.

Apelin-APLNR signaling contributes to the maintenance of muscle metabolism and function. Experimental studies indicate that it promotes mitochondrial biogenesis, autophagy, and anti-inflammatory responses in muscle fibers. Apelin also acts on muscle stem cells, enhancing their regenerative capacity. Both muscular apelin production and APLNR expression have been reported to decrease with aging, whereas deficiency of either apelin or its receptor exacerbates the age-dependent decline in muscle function in mice. Restoration of apelin signaling improves muscle function, mitochondrial homeostasis, and regenerative capacity in aged animals [41,87].

Overall, muscle-derived apelin can be regarded as a contraction-responsive myokine involved in experimental models in the preservation of mitochondrial homeostasis, metabolic function, regenerative capacity, and muscle performance. Its decline with aging may contribute to age-related muscle dysfunction, while restoration of apelin signaling represents a potential therapeutic approach. Nevertheless, most causal evidence derives from animal models, and the clinical efficacy and safety of targeting the apelin–APLNR pathway in older humans remain to be established. Moreover, circulating apelin is not necessarily a specific biomarker of muscular apelin production or sarcopenia.

- ***Meteorin-like (METRNL)***

Meteorin-like (METRNL), also known as meteorin-β, is a secreted factor produced by skeletal muscle, adipose tissue, and immune cells, particularly macrophages [20,28,41,85,88,89]. Muscular METRNL mRNA expression increases following selected forms of exercise, including high-intensity interval exercise, supporting its classification as an exercise-responsive myokine [88,90]. However, the human studies primarily demonstrated transcriptional regulation, and direct net secretion of METRNL from exercising human muscle has not been established. Skeletal muscle is not its exclusive source, and changes in circulating METRNL cannot automatically be attributed to muscular secretion [20,85,88].

METRNL was initially proposed to mediate communication between skeletal muscle and adipose tissue. In mice, increased METRNL promoted an immune-mediated thermogenic program in white adipose tissue, increased energy expenditure, and improved glucose homeostasis [88]. METRNL also contributes to skeletal muscle regeneration. Following muscle injury, it promotes a reparative macrophage phenotype and stimulates macrophage-derived IGF-1, which subsequently enhances satellite-cell proliferation and supports muscle repair. These effects involve STAT3 activation in macrophages and the subsequent production of IGF-1, which acts directly on primary muscle satellite cells [89]. Importantly, infiltrating immune cells (and macrophages in particular) appear to be a major functionally relevant source of METRNL in injured muscle. The regenerative actions observed in this setting should therefore not be attributed specifically to myofiber-derived METRNL.

Overall, METRNL can be regarded as an exercise- and stress-responsive myokine and, more broadly, as an injury-responsive mediator within the muscle microenvironment involved in communication between skeletal muscle, immune cells, and adipose tissue. Its best-supported actions include regulation of reparative macrophages, promotion of macrophage-derived IGF-1-dependent muscle regeneration, and modulation of adipose-tissue thermogenesis and systemic metabolism. Nevertheless, most causal evidence derives from animal models, and the contribution of muscle-derived METRNL to circulating concentrations and metabolic adaptation in humans remains incompletely established.

- ***Musclin (Osteocrin)***

Musclin, also known as osteocrin, is a secreted peptide encoded by the *OSTN* gene and expressed predominantly in skeletal muscle and bone. Its muscular expression increases in response to physical activity. Activity-induced musclin production involves calcium-dependent activation of Akt1, which relieves FoxO1-mediated repression of *OSTN* transcription, supporting its classification as an exercise-responsive myokine. Nevertheless, skeletal muscle is not its exclusive tissue source [91].

Musclin has structural similarity to natriuretic peptides but does not directly activate the guanylyl-cyclase-coupled natriuretic peptide receptors NPR-A or NPR-B. Instead, it binds to NPR-C and can reduce natriuretic peptide clearance, thereby increasing the availability of biologically active natriuretic peptides and potentiating their signaling. In experimental models, this enhances cyclic GMP-dependent signaling, mitochondrial biogenesis, oxidative capacity, and exercise endurance. Mice lacking musclin exhibit impaired exercise tolerance, whereas recombinant musclin restores physical performance [91]. Muscle-derived musclin may also support cardiovascular adaptation and protect the heart during pathological overload, although these systemic effects have been demonstrated mainly in animal models [92].

Overall, muscle-derived musclin can be regarded as an activity-responsive myokine with predominantly local actions and a potential endocrine role that facilitates skeletal muscle adaptation by enhancing natriuretic peptide signaling, mitochondrial remodeling, and oxidative capacity. Its best-supported physiological function in experimental models is the improvement of exercise tolerance, whereas its effects on cardiovascular protection remain more context-dependent. Most causal evidence derives from experimental models, and the contribution of musclin to exercise adaptation and metabolic regulation in humans remains incompletely established.

- ***Vascular Endothelial Growth Factor A (VEGF-A)***

VEGF-A is a key signaling protein and the principal pro-angiogenic member of the VEGF family. It can be considered a predominantly paracrine myokine. Skeletal muscle fibers express VEGF-A and release it into the muscle interstitium in response to contraction. Its expression is also stimulated by other factors such as hypoxia, increased metabolic demand, and exercise-related pathways. Once released, muscle-derived VEGF-A acts mainly on adjacent endothelial cells through VEGF receptor 2, promoting their survival, proliferation, migration, and organization into new capillary structures. Through these endothelial actions, VEGF-A helps adapt the local microvascular supply to the metabolic requirements of contracting muscle [20,28,93].

Through these actions, VEGF-A plays a central role in maintaining the skeletal muscle microvasculature and in the angiogenic adaptation to exercise and training. Experimental deletion of VEGF-A from myocytes reduces muscle capillarity, prevents normal training-induced capillary growth, and blunts training-related improvements in exercise performance, demonstrating that myofiber-derived VEGF-A is functionally relevant rather than merely expressed in muscle [94].

Overall, muscle-derived VEGF-A can be regarded as a predominantly paracrine myokine that coordinates skeletal muscle metabolic demands with the development and maintenance of an adequate microvascular supply. Its best-established actions include endothelial-cell survival, proliferation and migration, and exercise-induced angiogenesis. Insufficient muscle-derived VEGF-A activity may lead to reduced capillarity, impaired training-induced angiogenesis, and diminished exercise adaptation. Its appropriate spatial and temporal regulation is therefore important for matching the muscle microvascular supply to local metabolic requirements.

- ***Leukemia Inhibitory Factor (LIF)***

LIF is a pleiotropic member of the interleukin-6 cytokine family and a well-established contraction-regulated myokine. LIF binds to a heterodimeric receptor complex formed by its specific receptor and the common signal-transducing subunit gp130. Receptor activation engages several intracellular pathways, particularly JAK/STAT3, but also MAPK/ERK and PI3K/Akt signaling, thereby regulating myogenic-cell survival, proliferation, and differentiation [20,41]. Skeletal muscle expression of LIF increases in response to contractile activity. In humans, a single bout of heavy resistance exercise produced an approximately nine-fold increase in muscular LIF mRNA, while LIF remained undetectable in plasma. Moreover, electrically stimulated primary human myotubes produced and secreted LIF. These observations indicate that contraction-induced LIF acts predominantly within the local muscle environment rather than as a conventional circulating hormone. Its production may be regulated by pathways involving PI3K, Akt, and mTOR [22].

The best-established action of muscle-derived LIF is the regulation of the myogenic-cell population. This myokine induces the expression of proliferation-associated transcription factors, including JunB and c-Myc, and stimulates the proliferation of human myoblasts, potentially expanding the population of myogenic progenitors available for muscle adaptation and repair [22]. Although increased survival has also been reported in some experimental systems, the human contraction model primarily demonstrates stimulation of myoblast proliferation. Evidence from animal models also supports a role for LIF in skeletal muscle regeneration. Regeneration after muscle injury is impaired in LIF-deficient mice, whereas local administration of LIF stimulates the regenerative response [95]. These findings demonstrate a non-redundant role for LIF in the experimental injury model, although they do not establish that LIF alone controls regeneration. LIF also appears to contribute to load-induced hypertrophy: mice lacking LIF show an absent hypertrophic response in the predominantly fast plantaris muscle and a delayed response in the soleus, which can be restored by exogenous LIF administration [96]. The effect is therefore muscle- or fiber-type-dependent rather than uniformly absent in all overloaded muscles. LIF may also regulate the transition between myoblast expansion and differentiation. Experimental studies indicate that sustained LIF exposure can inhibit early myogenic differentiation and myotube formation through ERK-dependent mechanisms. In cultured C2C12 cells, this inhibitory effect occurred when LIF was administered during the early phase after induction of differentiation. This effect may be physiologically useful during the initial phase of regeneration by maintaining an adequate pool of undifferentiated progenitors. Subsequent attenuation of LIF signaling may then be required to permit differentiation and fusion into regenerating fibers. The latter interpretation remains a mechanistic model rather than a fully demonstrated temporal sequence in humans [97].

Overall, muscle-derived LIF can be regarded as a contraction- and injury-responsive autocrine/paracrine myokine that primarily regulates the balance between expansion and differentiation within the myogenic progenitor cell population. Its best-established functions include stimulation of myoblast proliferation, support of skeletal muscle regeneration, and contribution to load-induced hypertrophy. LIF signaling is highly context-dependent: transient and locally restricted activation appears to facilitate muscle adaptation and repair, whereas different concentrations and durations of exposure may produce distinct or even opposing biological responses. The biological consequences of LIF therefore depend on its cellular source, local concentration, duration of exposure, and timing within the regenerative process.

- ***Brain-derived neurotrophic factor (BDNF)***

BDNF is a member of the neurotrophin family that is expressed not only in the nervous system but also in skeletal muscle. It is initially synthesized as a precursor (proBDNF), which can subsequently be cleaved to generate mature BDNF. These molecular forms may exert different biological effects. While mature BDNF preferentially activates the tropomyosin receptor kinase B (TrkB), proBDNF can signal through complexes containing the low-affinity neurotrophin receptor p75NTR. Consequently, the biological actions attributed to BDNF depend not only on its concentration but also on its molecular form, receptor availability, cellular source, and physiological context [98]. Skeletal muscle fibers, myoblasts, and satellite cells can express BDNF, and muscle contraction, exercise, and tissue injury may modify its expression. Early studies showed that electrically induced contraction increased BDNF production in cultured muscle cells and that exercise enhanced its expression in human skeletal muscle [99,100]. Those early studies did not detect net release of muscle-derived BDNF into the circulation, supporting predominantly autocrine or paracrine actions within the muscle microenvironment. More recent isoform-specific evidence, however, indicates that this conclusion depends on the molecular form and exercise conditions: proBDNF, rather than mature BDNF, may be the predominant form expressed in skeletal muscle, and its abundance increases after exercise. Arteriovenous measurements have also demonstrated net release of proBDNF (but not mature BDNF) from exercising muscle following high-intensity exercise. Thus, although BDNF is commonly classified as a myokine, the precise contribution of skeletal muscle to circulating mature BDNF remains uncertain, whereas skeletal muscle may contribute to circulating proBDNF under selected exercise conditions [98,101].

One of the best-characterized actions of muscle-derived BDNF is the regulation of energy metabolism. In cultured myotubes and experimental muscle models, BDNF activates AMP-activated protein kinase and increases the phosphorylation and inhibition of acetyl-CoA carboxylase, thereby promoting fatty-acid oxidation. Muscle-derived BDNF may therefore facilitate the adaptation of substrate utilization to increased energy demand [100]. BDNF also participates in the regulation of satellite cells and muscle regeneration. It is expressed by muscle progenitor cells and contributes to the normal regulation of their proliferation and differentiation. Experimental reduction or deletion of muscle-derived BDNF alters the balance between satellite-cell proliferation and differentiation and impairs early muscle regeneration. In muscle-specific BDNF-deficient mice, Pax7 expression was reduced, the induction of differentiation markers was delayed, and regenerating fibers appeared later after injury. Restoration or addition of BDNF rescued several of these abnormalities. BDNF should therefore be regarded as a regulator required for normal myogenic progression rather than simply as an inhibitor of differentiation [99].

Overall, muscle-derived BDNF appears to coordinate several components of skeletal muscle adaptation, including lipid oxidation, myogenic-cell regulation, and regeneration. Its actions are nevertheless highly context-dependent. Transient and locally regulated BDNF signaling may support metabolic adaptation and tissue repair, whereas differences in the relative abundance of proBDNF and mature BDNF or altered signaling through TrkB and p75NTR may produce different or even opposing effects. BDNF should therefore be regarded as a multifunctional, predominantly local myokine rather than simply as an exercise-induced endocrine factor, although recent evidence supports an isoform- and exercise-intensity-dependent release of proBDNF from exercising muscle.

- ***Follistatin-like protein 1 (FSTL1)***

FSTL1 is a secreted extracellular glycoprotein that contains a follistatin-like domain and can act through autocrine, paracrine, and endocrine mechanisms. Although structurally related to follistatin, FSTL1 should not be regarded as a functional equivalent of this protein. Its biological actions are context-dependent and include signaling through Akt, endothelial nitric oxide synthase, and AMP-activated protein kinase in vascular target cells. FSTL1 can be classified as a *bona fide* myokine because primary human skeletal muscle cells express and secrete it. Its production changes during myogenic differentiation and is stimulated in cultured human myotubes by inflammatory mediators such as IL-1β and interferon-γ. Acute exercise has also been associated with an increase in circulating FSTL1, although this observation alone does not establish that skeletal muscle is the exclusive source of the circulating protein. Moreover, electrical pulse stimulation did not increase FSTL1 expression or secretion in primary human myotubes, indicating that its exercise-associated regulation should not be assumed to result directly from contractile activity. Muscle FSTL1 expression is additionally increased by Akt signaling, ischemia, hypoxia, and several forms of exercise in experimental models [102,103].

The best-established function of muscle-derived FSTL1 is the regulation of the local vasculature. FSTL1 promotes endothelial-cell survival, migration, and organization into vascular structures and enhances revascularization of ischemic skeletal muscle. These actions involve activation of Akt and endothelial nitric oxide synthase (eNOS), resulting in increased nitric oxide signaling [103]. FSTL1 may therefore help coordinate increases in muscle size or metabolic demand with an adequate expansion of the capillary network. Muscle-derived FSTL1 may also exert endocrine effects on the cardiovascular system. Experimental manipulation of FSTL1 expression specifically in skeletal muscle alters the vascular response to arterial injury. Muscle-specific deletion of FSTL1 increases neointimal formation after arterial injury, whereas muscular overexpression attenuates this response. These effects are associated with regulation of vascular smooth-muscle-cell proliferation through an AMPK-dependent mechanism [104]. However, because the heart, vascular cells, adipose tissue, and other organs also produce FSTL1, the contribution of skeletal muscle to its systemic actions must be established separately in each experimental context. By contrast, the direct actions of FSTL1 on skeletal muscle fibers, satellite cells, and myogenic regeneration remain less clearly defined. Although FSTL1 expression and secretion change during the differentiation of human myogenic cells, this observation does not by itself establish a direct role in controlling myoblast differentiation or muscle regeneration. At present, there is insufficient evidence to state that FSTL1 directly stimulates muscle hypertrophy or satellite-cell-mediated regeneration in the same well-established manner as classical myogenic growth factors [102].

Overall, FSTL1 can be regarded as an exercise-associated and stress-responsive myokine whose most firmly established functions in experimental models involve endothelial protection, angiogenesis, and communication between skeletal muscle and the cardiovascular system. Its direct roles in myogenic differentiation, hypertrophy, and regeneration remain less completely characterized. The effects of FSTL1 are likely to depend on its cellular source, concentration, target-cell signaling context, and the physiological or pathological setting.

- ***Locally produced Insulin-like Growth Factor 1 (IGF-1)***

IGF-1 is a peptide growth factor with major roles in skeletal muscle growth, maintenance, and regeneration. Although the liver is an important source of circulating endocrine IGF-1, skeletal muscle also produces this factor locally. Muscle-derived IGF-1 acts predominantly through autocrine and paracrine mechanisms on myofibers, satellite cells, myoblasts, and other components of the local tissue environment. In this context, locally produced IGF-1 can be considered a muscle-derived growth factor and, according to a broad definition, a myokine. However, circulating IGF-1 should not be assumed to originate from skeletal muscle [20,41]. The expression of the *IGF1* gene in skeletal muscle is regulated by mechanical loading, contractile activity, growth hormone, nutritional status, and tissue injury. Alternative splicing generates several IGF-1 transcripts that encode a common mature IGF-1 peptide but differ in their signal peptides and carboxy-terminal E domains. In human skeletal muscle, resistance exercise can modify the expression of these splice variants. In particular, an early increase in the transcript commonly designated IGF-1Ec or mechano-growth factor (MGF, see below for more details) has been reported after high-resistance exercise, although this response appears to be attenuated with aging [105]. MGF should, however, be understood primarily as the designation of an alternatively spliced transcript and its corresponding pro-peptide, rather than as evidence for an independently secreted mature growth factor. The physiological relevance of the different transcripts may involve differences in their processing, stability, localization, or temporal expression. Nevertheless, the existence and independent secretion of an endogenous E-domain peptide with biological actions distinct from mature IGF-1 remain controversial [41]. At the transcriptional level, however, MGF/IGF-1Ec mRNA increased 2.5 h after resistance exercise in younger, but not older, participants, without a corresponding change in IGF-1Ea mRNA [105].

Most of the established actions of locally produced IGF-1 are mediated by its tyrosine kinase receptor, which is expressed by muscle fibers and myogenic progenitor cells. IGF-1 receptor activation recruits intracellular signaling pathways that include phosphoinositide 3-kinase (PI3K) and Akt. Akt subsequently activates mTOR-dependent signaling and inhibits glycogen synthase kinase 3 (GSK-3), thereby stimulating translation and promoting myotube hypertrophy [106]. IGF-1-Akt signaling also inhibits FoxO transcription factors, reducing the induction of the muscle-specific ubiquitin ligases atrogin-1/MAFbx and MuRF1 and suppressing catabolic signaling [107]. IGF-1 can therefore favor muscle growth by simultaneously increasing protein synthesis and limiting protein degradation. These effects on atrophy-related genes have been demonstrated in experimental catabolic models and should not be interpreted as uniform suppression of proteolysis under every physiological condition. Local IGF-1 signaling is a potent inducer of skeletal muscle hypertrophy. Muscle-restricted expression of a locally acting IGF-1 isoform produces sustained increases in muscle mass and strength in mice [108]. Part of this response involves activation and incorporation of satellite-cell-derived nuclei into growing fibers. The relative contribution of satellite cells and direct anabolic signaling within differentiated myofibers is likely to vary with the experimental model, duration of stimulation, and extent of tissue remodeling [41,108]. IGF-1 also has a central role in muscle regeneration. It promotes the activation, survival, and proliferation of satellite cells and supports their subsequent progression through the myogenic program. Local expression of muscle-restricted IGF-1 accelerates regeneration after experimental injury, facilitates the formation and growth of regenerating fibers, and helps preserve regenerative capacity in aged muscle [41,108]. In this regard, aged mice expressing a locally acting, muscle-restricted IGF-1 transgene showed sustained functional hypertrophy and preserved proliferative and regenerative responses to muscle injury [108].

These effects are not limited to myogenic cells. Muscle-derived IGF-1 can also modify the tissue environment by regulating inflammatory-cell recruitment, reducing prolonged expression of pro-inflammatory mediators, and limiting fibrosis. Through these combined actions, IGF-1 helps coordinate the expansion of muscle progenitors with resolution of inflammation and restoration of tissue architecture [109]. In a muscle-restricted transgenic model, these changes included reduced TNF-α and IL-1β expression, altered monocyte/macrophage-associated chemokine expression, and reduced fibrotic remodeling.

The actions of IGF-1 on myogenic cells are stage-dependent. During the initial phases of repair, IGF-1 promotes satellite-cell and myoblast proliferation and increases the number of progenitors available for regeneration. At later stages, it supports myogenic differentiation, myotube formation, protein accretion, and maturation of newly formed fibers. These apparently different effects are influenced by the timing and concentration of IGF-1 exposure, the differentiation state of the target cell, receptor abundance, and the local availability of IGF-binding proteins [41]. IGF-1 additionally contributes to muscle-cell survival. Activation of Akt inhibits several pro-apoptotic and catabolic pathways and may protect muscle fibers and progenitor cells during mechanical stress, denervation, inflammation, or metabolic challenge [41,107]. Local IGF-1 expression has therefore been investigated as a potential means of limiting muscle loss in aging, disuse, neuromuscular disease, and cachexia. Nevertheless, experimental results are not uniform. The consequences of muscular IGF-1 overexpression vary according to the isoform, promoter, degree of expression, leakage into the circulation, age, and injury model. Consequently, the benefits observed with a locally restricted IGF-1 construct should not be generalized to every form of muscular or systemic IGF-1 administration [20,41].

Overall, locally produced IGF-1 can be regarded as a predominantly autocrine and paracrine myokine that links mechanical and tissue-damage signals to muscle growth and repair. Its best-established actions include stimulation of protein synthesis, attenuation of atrophy-associated catabolic signaling, regulation of satellite cells and myogenic differentiation, promotion of muscle-fiber hypertrophy, and support of regeneration. However, these actions are highly dependent on the spatial and temporal characteristics of IGF-1 signaling, and the biological significance of individual IGF1 splice variants and their proposed independent E-domain products requires careful interpretation.

- *Mechano Growth Factor (MGF)/IGF-1Ec*

As previously mentioned, IGF-1Ec (commonly referred to as MGF) is a mechanically responsive *IGF1* splice variant expressed in skeletal muscle. It may be regarded, in a broad sense, as a putative autocrine/paracrine muscle-derived growth signal. However, the available evidence principally demonstrates regulation of the *IGF-1Ec* transcript and its corresponding pro-IGF-1 isoform; whether an endogenous Ec-domain peptide is physiologically generated and secreted and exerts biological effects independently of mature IGF-1 remains controversial [41,105]. Interestingly, increased *MGF* mRNA expression together with increased *IGF1* expression and markers of satellite-cell activation was observed in the external intercostal muscles of patients with severe chronic obstructive pulmonary disease (COPD) [110], which is compatible with ongoing cycles of muscle microdamage and repair. However, these findings demonstrate regulation of the transcript but do not establish the independent production or secretion of an Ec-domain peptide.

### 2.1. Other Context-Dependent Myokines and Muscle-Derived Factors

- ***Fibroblast Growth Factor 21 (FGF21)***

FGF21 is a secreted member of the endocrine FGF subfamily that plays an important role in the regulation of energy metabolism and cellular adaptation to stress. Although the liver is the principal source of circulating FGF21 under most physiological conditions, skeletal muscle can also express and secrete this growth factor. Muscle-derived FGF21 may therefore act through autocrine or paracrine mechanisms within skeletal muscle and, when released in sufficient amounts, as an endocrine myokine affecting distant tissues. However, FGF21 expression is very low in healthy resting muscle, and it is more appropriately regarded as an inducible stress myokine or myomitokine rather than as a constitutively secreted muscle factor [20,28,111,112].

Muscular FGF21 expression is induced by several conditions that disturb cellular and mitochondrial homeostasis, including impaired mitochondrial fatty-acid oxidation, respiratory-chain dysfunction, mitochondrial uncoupling, endoplasmic-reticulum stress, fasting, denervation, and some forms of contractile activity. Many of these stimuli activate the integrated stress response, particularly pathways involving eukaryotic initiation factor 2α (eIF2α) and the transcription factor ATF4 [20,28,111,112,113]. These observations demonstrate that skeletal muscle is capable of producing and releasing FGF21, although the evidence derives predominantly from cellular and animal models. In humans, acute exercise can increase circulating FGF21, but arteriovenous measurements have demonstrated increased splanchnic FGF21 secretion without detectable net release from the exercising leg, suggesting that the exercise-induced systemic response may arise predominantly from the liver rather than skeletal muscle [114]. Canonical FGF21 signaling requires a fibroblast growth factor receptor, most commonly FGFR1c, together with the obligate co-receptor β-Klotho. Activation of this receptor complex can engage ERK1/2 and other intracellular pathways involved in metabolic regulation. However, β-Klotho expression in skeletal muscle is relatively low. This low expression raises uncertainty regarding the magnitude of direct FGF21 signaling in normal adult muscle. Consequently, some reported direct effects of FGF21 on muscle may occur only under conditions in which the receptor complex is sufficiently expressed, and the responsiveness of normal human skeletal muscle to physiological concentrations of FGF21 remains incompletely defined [20,28,111].

FGF21 also participates in mitochondrial quality control and the regulation of muscle mass. Muscle-specific deletion of Fgf21 has little effect on muscle size or phenotype under basal conditions, indicating that FGF21 is not essential for normal muscle maintenance. During fasting, however, muscle-derived FGF21 promotes the expression of the mitophagy-related protein BNIP3 and facilitates the removal of mitochondria. Excessive or sustained activation of this pathway can increase autophagic degradation and contribute to muscle wasting. Accordingly, muscular overexpression of FGF21 induces loss of muscle mass, whereas muscle-specific Fgf21 deletion partially protects against fasting-induced atrophy. FGF21 may therefore mediate an adaptive mitochondrial recycling response during short-term nutrient deprivation but become catabolic when its expression is excessive or prolonged [112]. Muscle-derived FGF21 can also mediate communication with distant metabolic tissues. In mouse models of skeletal muscle mitochondrial uncoupling, increased muscular FGF21 expression raises circulating FGF21 and promotes remodeling and browning of white adipose tissue. Genetic deletion studies confirmed that FGF21 is required for this adipose-tissue response. However, the same experiments showed that many other adaptations to muscle mitochondrial stress, including improved glycemic control, resistance to diet-induced obesity, and activation of the muscle mitochondrial stress response, were largely preserved in the absence of this myokine. Thus, FGF21 contributes to specific components of muscle-to-adipose communication but should not be considered the sole mediator of systemic adaptation to muscular mitochondrial stress [111].

Recent evidence further emphasizes the potentially detrimental effects of locally produced FGF21 in pathological conditions. Following denervation, FGF21 is strongly induced in skeletal muscle and acts locally to promote loss of neuromuscular-junction innervation and muscle atrophy. Muscle-specific silencing of FGF21 preserved neuromuscular innervation and reduced denervation-induced wasting, whereas forced muscular expression produced the opposite effect. This response involved transforming growth factor β1 released by fibro-adipogenic progenitors, activation of the JNK/c-Jun pathway, and alterations in histone deacetylase 4 signaling. These findings identify muscle-derived FGF21 as a possible mediator, rather than merely a biomarker, of neurogenic muscle wasting. However, this mechanism has so far been demonstrated principally in experimental models [113].

Overall, locally produced FGF21 can be regarded as a stress-responsive myokine that links disturbances in muscle energy metabolism and mitochondrial function to local and systemic responses. Its actions in experimental models include regulation of mitophagy, muscle mass, adipose-tissue remodeling, and neuromuscular-junction innervation. These effects are strongly context-dependent: transient FGF21 induction may participate in selected metabolic and mitochondrial stress responses, whereas chronic or excessive muscular FGF21 signaling may promote excessive mitophagy or autophagic degradation, denervation-associated changes, and muscle wasting. FGF21 should therefore not be classified as intrinsically beneficial or detrimental, but as a marker and mediator of the muscular stress response whose effects depend on its source, concentration, duration, and target-tissue sensitivity.

- ***Hepatocyte Growth Factor (HGF)***

HGF, also known as scatter factor, is a pleiotropic growth factor with an important role in skeletal muscle growth and regeneration. HGF binds to MET (c-Met), a receptor tyrosine kinase expressed by quiescent and activated muscle satellite cells. Activation of the HGF-MET axis engages intracellular pathways that include MAPK/ERK and phosphoinositide 3-kinase–Akt signaling and regulates cell-cycle entry, migration, and myogenic progression [115]. HGF is constitutively present in adult skeletal muscle, where it has been localized to the extracellular matrix surrounding muscle fibers. Following mechanical stretch or tissue injury, matrix-bound HGF is rapidly released and becomes available to bind MET on adjacent satellite cells. Experimental studies indicate that this process involves calcium-calmodulin signaling, nitric oxide production, and activation of matrix metalloproteinases. This mechanism allows skeletal muscle to convert a mechanical or injury-related stimulus into an immediate regenerative signal without requiring de novo HGF synthesis [116]. Skeletal muscle cells can produce HGF locally. Its expression has been demonstrated in differentiated myotubes and satellite cell cultures, and conditioned media from muscle-cell cultures contain biologically active HGF [115]. These observations support local autocrine or paracrine signaling but do not establish the relative contribution of myofibers, satellite cells, and non-myogenic populations to HGF production in vivo. Consequently, the detection of HGF in whole-muscle tissue does not by itself demonstrate its myofiber origin. Thus, HGF can be classified as a locally acting myokine when its muscular or myogenic cellular origin has been demonstrated, but it is more broadly regarded as a local muscle growth factor [20,41,115].

The best-established action of HGF in skeletal muscle is the activation of quiescent satellite cells, which subsequently enter the cell cycle, thereby initiating the regenerative response after mechanical loading or muscle injury. Direct administration of HGF to uninjured muscle is sufficient to induce satellite-cell activation, confirming that HGF is not merely associated with regeneration but can function as an initiating signal [116]. This myokine subsequently promotes the expansion and migration of myogenic progenitor cells. In primary satellite-cell and myoblast cultures, HGF increases DNA synthesis and cell proliferation and acts as a potent chemotactic factor [115,117]. HGF-MET signaling also enables activated myogenic cells to migrate through the injured tissue and reach areas requiring repair. Genetic or experimental disruption of MET signaling reduces myoblast motility, produces abnormalities in myocyte fusion, and impairs adult muscle regeneration. Thus, the HGF-MET axis participates not only in the initial activation of satellite cells but also in their spatial organization and incorporation into regenerating fibers [117]. The effects of HGF on myogenic differentiation are strongly dependent on timing and concentration. During the early regenerative phase, HGF maintains myoblasts in a proliferative state and can delay the expression of muscle-specific differentiation programs. This temporary inhibition of differentiation may be physiologically advantageous because it allows expansion of the progenitor-cell population before fusion and fiber formation occur. At later stages, however, attenuation or modification of HGF signaling appears necessary for terminal differentiation. Moreover, MET signaling itself contributes to efficient myocyte fusion, indicating that the relationship between HGF and differentiation is not simply inhibitory but varies according to the stage of the regenerative process [115,117]. In addition to its direct actions on myogenic cells, HGF can modify the inflammatory environment during muscle repair. In experimental muscle injury, HGF–MET signaling promotes the transition of infiltrating macrophages toward a reparative phenotype through pathways involving CaMKKβ and AMP-activated protein kinase. Pharmacological inhibition of MET altered macrophage phenotype-marker expression and delayed regeneration, whereas intramuscular HGF overexpression favored a reparative macrophage profile and facilitated tissue repair. These observations suggest that HGF coordinates muscle regeneration through simultaneous effects on satellite cells and immune cells [30,118].

Overall, locally available HGF acts as an early and tightly regulated mediator of skeletal muscle repair. Its best-supported functions include activation of quiescent satellite cells, stimulation of myogenic-cell proliferation and migration, temporal regulation of differentiation, contribution to myocyte fusion, and, in experimental models, modulation of the inflammatory environment. Its biological effects depend on its cellular source, release from the extracellular matrix, local concentration, duration of signaling, and stage of regeneration. HGF should therefore be regarded primarily as a local autocrine/paracrine growth factor rather than as a conventional circulating endocrine myokine.

- ***Growth Differentiation Factor 15 (GDF15)***

GDF15, also known as macrophage inhibitory cytokine 1, is a stress-responsive secreted protein distantly related to the TGF-β superfamily. Despite its name, GDF15 should not be regarded primarily as a conventional growth factor. Rather, it functions as a systemic signal of cellular and tissue stress. Its basal expression in healthy skeletal muscle is generally low, but it can increase markedly in response to mitochondrial dysfunction, impaired oxidative phosphorylation, endoplasmic-reticulum stress, inflammation, tissue injury, and some forms of contractile activity. Skeletal muscle cells are capable of producing and secreting GDF15. In experimental models, muscle-specific mitochondrial dysfunction activates a mitochondrial and integrated cellular stress response, leading to increased muscular GDF15 expression and elevated circulating concentrations. These findings have led to the classification of GDF15 as a stress-induced myokine or myomitokine [20,28,119]. In human primary myotubes, electrically induced contraction rapidly increased GDF15 gene expression and protein secretion. Acute exercise also increased GDF15 mRNA expression in human skeletal muscle. Nevertheless, the contribution of contracting skeletal muscle to circulating GDF15 during physiological exercise remains uncertain, because several other tissues can also release substantial amounts of this factor and net muscular release was not established in this study [120]. The best-characterized receptor for GDF15 is the GDNF family receptor α-like protein, GFRAL, which signals in association with the receptor tyrosine kinase RET. GFRAL expression is largely restricted to neurons in the area postrema and nucleus of the solitary tract in the hindbrain and has not been convincingly demonstrated in normal skeletal muscle. Activation of the GDF15-GFRAL-RET pathway reduces food intake, induces aversive responses, and modifies whole-body energy balance [121].

Thus, the most firmly established actions of circulating muscle-derived GDF15 are endocrine and centrally mediated rather than direct autocrine effects on the muscle fiber. In mouse models of muscle mitochondrial dysfunction, skeletal muscle-derived GDF15 contributes to a coordinated systemic metabolic response. GDF15 promotes reduced energy intake, adipose-tissue remodeling, metabolic flexibility, and improved insulin sensitivity. Genetic deletion of GDF15 abolished several of these systemic adaptations but did not prevent the muscle-cell-autonomous integrated stress response or mitochondrial-stress-induced muscle wasting. These observations indicate that GDF15 can communicate the presence of muscular mitochondrial stress to distant organs without necessarily correcting the underlying mitochondrial defect or acting directly on muscle mass [119]. Muscle-derived GDF15 may also participate in communication between skeletal muscle and adipose tissue. Conditioned media from electrically stimulated human myotubes increased lipolysis in human adipocytes, whereas neutralization of GDF15 attenuated this response. Recombinant GDF15 similarly stimulated glycerol and non-esterified fatty-acid release from human adipose-tissue explants. These results suggest that contraction-induced GDF15 may facilitate lipid mobilization and increase the availability of energetic substrates during exercise. However, because GFRAL is not expressed in adipose tissue, this proposed peripheral action would require a GFRAL-independent mechanism that has not yet been fully characterized [120,121]. GDF15 may also participate in muscle regeneration, although in this setting its principal local source may not be the muscle fiber itself. Following acute muscle injury, a specialized population of reparative macrophages produces GDF15. Deficiency of GDF15, including its loss from the myeloid compartment, delays the transition from inflammatory to reparative macrophage phenotypes and impairs the recovery of regenerating muscle fibers. Recombinant GDF15 also promotes the proliferation of primary myogenic progenitor cells. These findings support a local role for GDF15 in coordinating regenerative inflammation and myoblast expansion. However, because the factor is produced predominantly by infiltrating macrophages in this context, it would be more precise to describe it as a muscle-tissue-derived paracrine mediator than as a myofiber-derived myokine [30,122].

Overall, locally produced GDF15 can be regarded as a stress-responsive myokine when its synthesis and secretion by skeletal muscle cells have been demonstrated. Its most firmly established functions involve signaling muscular stress to the brain and contributing to the regulation of food intake, energy balance, adipose-tissue metabolism, and systemic adaptation. Within injured muscle, GDF15 produced by reparative macrophages may support regenerative inflammation and myogenic-progenitor proliferation, but this action should be distinguished from myofiber-derived endocrine signaling. Because the canonical GDF15 receptor has not been convincingly demonstrated in normal skeletal muscle, proposed direct autocrine actions should be interpreted cautiously until the responsible receptors and signaling pathways have been identified.

### 2.2. Inflammation-Responsive Cytokines

- ***Interleukin-1β (IL-1β)***

IL-1β is a potent pro-inflammatory cytokine that can be produced by skeletal muscle cells under conditions of injury, infection, metabolic stress, or chronic inflammation. IL-1β is synthesized as an inactive precursor, pro-IL-1β, whose conversion into the mature secreted cytokine generally requires inflammasome-dependent activation of caspase-1. Cultured myoblasts and myotubes possess functional inflammasome machinery and can produce IL-1β after combined inflammatory and purinergic stimulation [123]. However, macrophages and other infiltrating or resident cells may contribute substantially to IL-1β production within injured muscle. Thus, IL-1β should be classified as a myokine only when its production and release by myogenic cells have been specifically demonstrated. IL-1β signals through interleukin-1 receptor type 1 (IL-1R1), activating NF-κB- and MAPK-dependent pathways [124].

During the early response to acute muscle injury, transient IL-1 signaling can promote satellite-cell proliferation and coordinate interactions between inflammatory cells and myogenic progenitors. Experimental loss of IL-1α and IL-1β delays muscle regeneration, whereas IL-1β directly increases the proliferative activity of primary satellite cells. These findings suggest that local IL-1 signaling contributes to progenitor-cell expansion and the initiation of repair, with IL-1β exerting direct effects on myogenic cells in culture [125].

By contrast, sustained IL-1β exposure can adversely affect mature muscle fibers. In cultured myotubes, IL-1β increases the expression of the ubiquitin ligases atrogin-1/MAFbx and MuRF1, reduces sarcomeric actin abundance, and decreases myotube size [124].

Overall, muscle-cell-derived IL-1β can be regarded as an injury-, inflammation-, and metabolic-stress-responsive autocrine/paracrine myokine. Transient local IL-1 signaling, including direct IL-1β actions on myogenic cells, may support satellite-cell proliferation and the early phases of regeneration, whereas excessive or prolonged IL-1β exposure can promote muscle catabolism. Its biological consequences therefore depend on its cellular source, inflammasome activation, local concentration, duration of exposure, and the stage of the injury-repair response.

- ***Tumor Necrosis Factor α (TNF-α)***

TNF-α, currently also designated simply as TNF, is a pleiotropic pro-inflammatory cytokine that can be expressed and released by myoblasts and myotubes under selected inflammatory and myogenic conditions [126,127]. However, macrophages and other immune or stromal cells are frequently the predominant sources of TNF-α within injured or diseased muscle [30,128]. Moreover, contracting human skeletal muscle does not appear to release significant amounts of TNF-α during physiological exercise [129]. Thus, TNF-α should be classified as a myokine only when its production by muscle cells has been specifically demonstrated and is better regarded as an inflammation- or injury-responsive factor than as a conventional exercise-induced myokine [29,129,130]. Consistent with a role in human muscle remodeling, TNFR1 and TNFR2 expression in the external intercostal muscle of patients with severe COPD was positively associated with markers of myogenic repair, while TNFR2 expression was also related to inspiratory muscle function [131]. TNF-α signals through TNF receptor 1 and TNF receptor 2 (TNFR1 and TNFR2), activating pathways that include NF-κB, MAPK/p38, JNK, and mechanisms regulated by reactive oxygen species.

The effects of TNF-α on muscle regeneration are strongly dependent on timing and signal intensity. Low-level or transient TNF-α signaling during the response to acute injury promotes p38-dependent myogenic differentiation and contributes to the recovery of muscle structure and function. Mice lacking both TNFR1- and TNFR2-mediated signaling show impaired p38 activation, myogenesis, and regeneration, indicating that a controlled TNF-α response forms part of the normal injury-repair program [126,128,130]. Conversely, higher experimental TNF-α concentrations inhibit myogenic differentiation, demonstrating that its effects are concentration-dependent rather than uniformly regenerative.

By contrast, sustained or excessive TNF-α exposure can inhibit myogenic differentiation and impair mature muscle fibers. In cultured myotubes, TNF-α induces reactive-oxygen-species-dependent NF-κB activation, promotes ubiquitin conjugation, reduces total protein and myosin heavy-chain abundance, and produces muscle-cell protein loss [127]. Sustained TNF-α exposure can also inhibit myogenic differentiation by disrupting IGF-1-dependent signaling [132]. In healthy humans, experimental TNF-α infusion impaired skeletal-muscle insulin signaling and glucose uptake, with altered IRS-1 signaling and reduced phosphorylation of Akt substrate 160, a regulator of GLUT4 translocation [133]. These effects provide a mechanistic link between chronic TNF-α exposure, muscle wasting, and metabolic dysfunction, although TNF-α is usually one of several interacting mediators in systemic disease.

Overall, muscle-cell-derived TNF-α can be regarded as an injury-, inflammation-, and metabolic-stress-responsive autocrine/paracrine myokine. Transient and locally regulated TNF-α signaling supports p38-dependent differentiation and muscle regeneration, whereas prolonged or excessive TNF-α signaling can suppress myogenesis, promote protein loss, impair insulin signaling, and contribute to muscle atrophy. Its biological consequences therefore depend on its cellular source, local concentration, receptor balance, duration of exposure, and stage of the injury-repair response.

Despite their structural diversity, the myokines and muscle-derived factors discussed above converge on a relatively limited set of intracellular signaling modules, while differing markedly in receptor usage, cellular targets, and biological context. Cytokines such as IL-6 and LIF prominently engage JAK/STAT signaling, whereas several chemokines act through CXCR or CCR G-protein-coupled receptors that regulate calcium-dependent, MAPK/ERK, and PI3K/Akt pathways. Growth- and remodeling-related factors frequently converge on PI3K/Akt/mTOR and MAPK signaling, while myostatin is distinguished by canonical SMAD2/3 signaling, and FGF21 production is characteristically induced by integrated stress-response pathways. AMPK also emerges repeatedly as a metabolic signaling node linking several myokines to substrate utilization and mitochondrial adaptation. This convergence may partly explain the substantial functional overlap among otherwise distinct factors. However, shared downstream pathways do not imply biological equivalence, since the final response is critically determined by cellular source, receptor distribution, local versus systemic exposure, concentration, timing, and the physiological or pathological setting.

An additional source of apparent inconsistency across the myokine literature is the experimental level at which individual effects have been demonstrated. In vitro models are valuable for identifying potential receptors, signaling pathways, and direct cellular actions, but frequently use simplified cellular systems or recombinant concentrations that may not reproduce the muscle microenvironment or physiological exposure. Animal loss- and gain-of-function models can provide stronger evidence of causality, although genetic deletion, sustained overexpression, pharmacological administration, or experimental injury may generate exposures and compensatory responses that differ substantially from human physiology. Human studies provide the greatest physiological relevance but are often observational, may rely on circulating concentrations of uncertain tissue origin, and rarely establish tissue-specific secretion or causality. Accordingly, confidence is greatest when mechanistic, animal, and human evidence converge, whereas effects demonstrated only at one experimental level should be considered provisional and should not be directly extrapolated to human physiology or disease.

## 3. Myokines in Disease and Aging: An Interpretative Framework

Although myokines contribute to physiological adaptation and inter-organ homeostasis, their production and signaling may be profoundly altered in disease. In these settings, skeletal muscle is not merely a target of systemic inflammation, metabolic dysfunction, hypoxia, inactivity, denervation, malnutrition, or treatment-related toxicity, but may also become an active contributor to the systemic response through changes in its secretory profile. Altered myokine profiles may therefore represent causal mediators of tissue dysfunction, compensatory attempts to restore homeostasis, consequences of tissue injury or systemic stress, or biomarkers reflecting disease severity [20,28,45,85,134].

Despite their different etiologies, muscle and neuromuscular disorders, chronic respiratory and other systemic diseases, cancer, and aging share several mechanisms that may alter myokine production and signaling [134,135,136]. These include persistent inflammation, impaired protein homeostasis, mitochondrial and metabolic stress, defective regeneration, fibrosis, vascular and neuromuscular alterations, and disrupted communication between skeletal muscle and distant organs. These shared mechanisms provide a useful framework for interpreting myokine changes across disease and aging, but their relative contribution—and therefore the biological significance of a given myokine—may vary substantially with disease-specific factors such as inflammation, hypoxia, insulin resistance, denervation, malignancy, aging, treatment exposure, and the amount and functional state of skeletal muscle [20,28,30,45,134,135,136].

Interpretation of disease-associated changes in myokines therefore requires several specific distinctions. First, an increase or decrease in circulating concentration should not be attributed to skeletal muscle without evidence of muscular production and release, because many candidate myokines are also produced by immune cells, adipose tissue, liver, cardiovascular tissues, tumors, and other organs [20,28,85]. Second, local autocrine or paracrine signaling within muscle should be distinguished from endocrine actions inferred from circulating concentrations, since these compartments may differ substantially in ligand concentration and biological relevance. Third, temporal context is critical: an acute increase associated with exercise, injury, or regeneration cannot be directly extrapolated to chronic disease, where sustained exposure may have different biological consequences. Fourth, receptor abundance and cell-specific receptor distribution determine whether changes in ligand concentration can translate into biological signaling. Finally, associations between circulating myokines and muscle mass, strength, functional impairment, or disease severity cannot by themselves establish causality, since altered concentrations may represent causal mediators, compensatory responses, or secondary markers of tissue stress [20,30,45,85].

Several experimental approaches can help resolve these uncertainties. Attribution of tissue origin can be strengthened by combining muscle-tissue measurements with circulating concentrations, assessing arteriovenous gradients across skeletal muscle, using tracer-based approaches where applicable, and applying tissue- and cell-specific approaches, including single-cell and spatial analyses and, in experimental models, muscle-specific loss- or gain-of-function strategies. Distinguishing local from systemic signaling requires assessment of the muscle microenvironment and target-tissue signaling together with circulating concentrations, rather than relying on plasma measurements alone. Serial sampling during exercise, injury and recovery, as well as longitudinal studies across disease progression or therapeutic intervention, is required to define the temporal relationship between myokine changes and phenotype. Receptor expression should likewise be assessed at the relevant cell-type level and, where possible, linked to activation of downstream signaling pathways. Finally, establishing causality requires interventions that modify the ligand, receptor, or downstream pathway and demonstrate corresponding changes in muscle or systemic outcomes; cross-sectional circulating associations should therefore be regarded primarily as hypothesis-generating.

## 4. Translational Implications: Myokines as Biomarkers and Therapeutic Targets

The growing recognition of the secretory and signaling functions of skeletal muscle has generated considerable interest in the potential use of myokines as biomarkers and therapeutic targets [20,28,41,85]. From a biomarker perspective, circulating or tissue-associated myokine profiles could theoretically provide information on muscle metabolic stress, regenerative activity, inflammatory status, mitochondrial dysfunction, or the progression of muscle wasting [20,28,85,134,135,136]. Such information may be relevant not only to primary muscle disorders but also to neurological, cardiovascular, respiratory, rheumatological, metabolic, malignant, and other systemic conditions in which skeletal muscle is secondarily affected [85,134,135,136]. However, translation into clinically useful biomarkers remains challenging. Many candidate myokines are produced by several tissues, and their circulating concentrations therefore do not necessarily reflect skeletal-muscle production [20,28,85]. Moreover, concentrations may vary with exercise, nutritional status, age, sex, body composition, inflammation, renal or hepatic function, circadian factors, and the timing of sampling relative to muscle activity or injury [28,85].

Analytical and biological heterogeneity represents an additional limitation. Different assays may detect distinct molecular forms, precursor proteins, processed peptides, or protein complexes, as illustrated particularly clearly by irisin, BDNF, myostatin, and related factors [69,70,75,77,98,101]. Consequently, tissue expression, circulating abundance, receptor engagement, and biological activity should not be regarded as interchangeable readouts. A change in circulating concentration does not, by itself, establish effective target-tissue exposure, receptor activation, or downstream signaling. Moreover, for many myokines, clinically meaningful reference ranges and disease-specific cut-off values remain undefined, and cross-sectional associations with muscle mass, strength, exercise capacity, or disease severity do not establish either muscular origin or causal involvement [20,69,70,85]. These considerations suggest that the greatest biomarker potential may ultimately reside not in individual circulating myokines but in disease- and context-specific signatures combining several secreted factors with conventional clinical variables, imaging, functional measurements, and potentially proteomic or transcriptomic information [20,85].

Myokine signaling also provides several potential therapeutic opportunities [20,41]. Conceptually, treatment may involve inhibiting a maladaptive signal, enhancing an adaptive or regenerative pathway, modifying receptor activation, or reproducing selected components of the muscle-secretory response to exercise. The most advanced examples illustrate both the promise and complexity of this approach. Targeting the myostatin pathway has long been pursued as a means of increasing muscle mass [41,75,76,77,78,79,80], although earlier studies also demonstrated that greater muscle mass does not necessarily translate into proportional improvements in strength or function [41,75]. More recently, selective inhibition of myostatin activation with apitegromab has been evaluated in a phase 3 trial in spinal muscular atrophy, illustrating the potential value of muscle-directed treatment as a complement to therapies acting on the primary neurological defect [137]. Similarly, neutralization of GDF15 with ponsegromab has produced improvements in body weight, appetite, cachexia symptoms, and physical activity in patients with cancer cachexia selected for elevated circulating GDF15 [138]. Importantly, in this setting GDF15 should not be interpreted specifically as a muscle-derived factor, since multiple tissues can contribute to its circulating levels [119,120,121,122]. Nevertheless, this study provides a clinically relevant example of how a molecule discussed within the broader myokine landscape can serve simultaneously as a mechanistically informed biomarker and therapeutic target.

Other myokine pathways remain at earlier stages of translational development. Modulation of apelin, IL-15, IGF-1, FGF21, inflammatory cytokines, chemokines, or regenerative growth factors has produced potentially favorable effects in experimental systems [41,42,44,46,63,87,108,109,111,112,113], but their pleiotropic biology raises substantial safety and specificity concerns [20,41,85]. A mediator that promotes regeneration, angiogenesis, or immune-cell recruitment when transiently activated within muscle may have undesirable consequences when administered systemically or chronically. Conversely, systemic inhibition of a cytokine associated with pathological muscle wasting could interfere with its physiological role in exercise adaptation, tissue repair, or host defense. Therapeutic development must therefore consider not only the identity of the target but also the cellular source, molecular form, receptor distribution, dose, route of administration, and temporal pattern of signaling [20,28,41,85]. Tissue-selective delivery or modulation of specific ligand-receptor interactions may ultimately prove more effective than generalized stimulation or inhibition of broadly active signaling pathways.

Several methodological advances will be required to move the field forward [20,28,85]. Human studies should increasingly incorporate longitudinal designs, standardized and molecular-form-specific assays, direct assessment of muscle production when feasible, and functional endpoints in addition to circulating concentrations. Arteriovenous measurements, tissue-specific approaches, single-cell and spatial analyses, and integrated proteomic profiling may help distinguish muscle-derived signals from molecules originating in other tissues and identify the cellular populations responsible for their production [20,28,69,98,101,114]. Intervention studies should determine whether changes in a candidate myokine precede and predict changes in muscle phenotype, whether manipulation of the pathway modifies clinically meaningful outcomes, and whether effects observed in cellular or animal models occur at physiological concentrations in humans.

Taken together, these considerations suggest a stepwise framework for the investigation and translation of candidate myokines. An initial association between circulating concentration and a physiological or clinical phenotype should first be followed by source-attribution studies demonstrating skeletal-muscle production and release. The next step is to establish target-tissue exposure, receptor engagement, and downstream signaling at physiologically relevant concentrations, followed by mechanistic studies showing that perturbation of the pathway modifies the relevant phenotype. Only thereafter should a candidate be advanced as a biomarker, requiring analytical validation, reproducibility, longitudinal association, and added clinical value in a defined context, or as a therapeutic target, requiring evidence that pathway modulation produces clinically meaningful benefit with acceptable safety.

Overall, the translational potential of myokines is substantial but highly heterogeneous [20,85]. Some pathways are beginning to progress from mechanistic biology toward biomarker-guided or targeted therapeutic strategies, whereas others remain primarily experimental. The most promising future approach is therefore unlikely to involve classifying myokines globally as beneficial or detrimental, but rather identifying specific molecular signatures and signaling pathways that are informative or therapeutically actionable in defined clinical and biological contexts.

## 5. Conclusions and Future Perspectives

**Figure 3:**
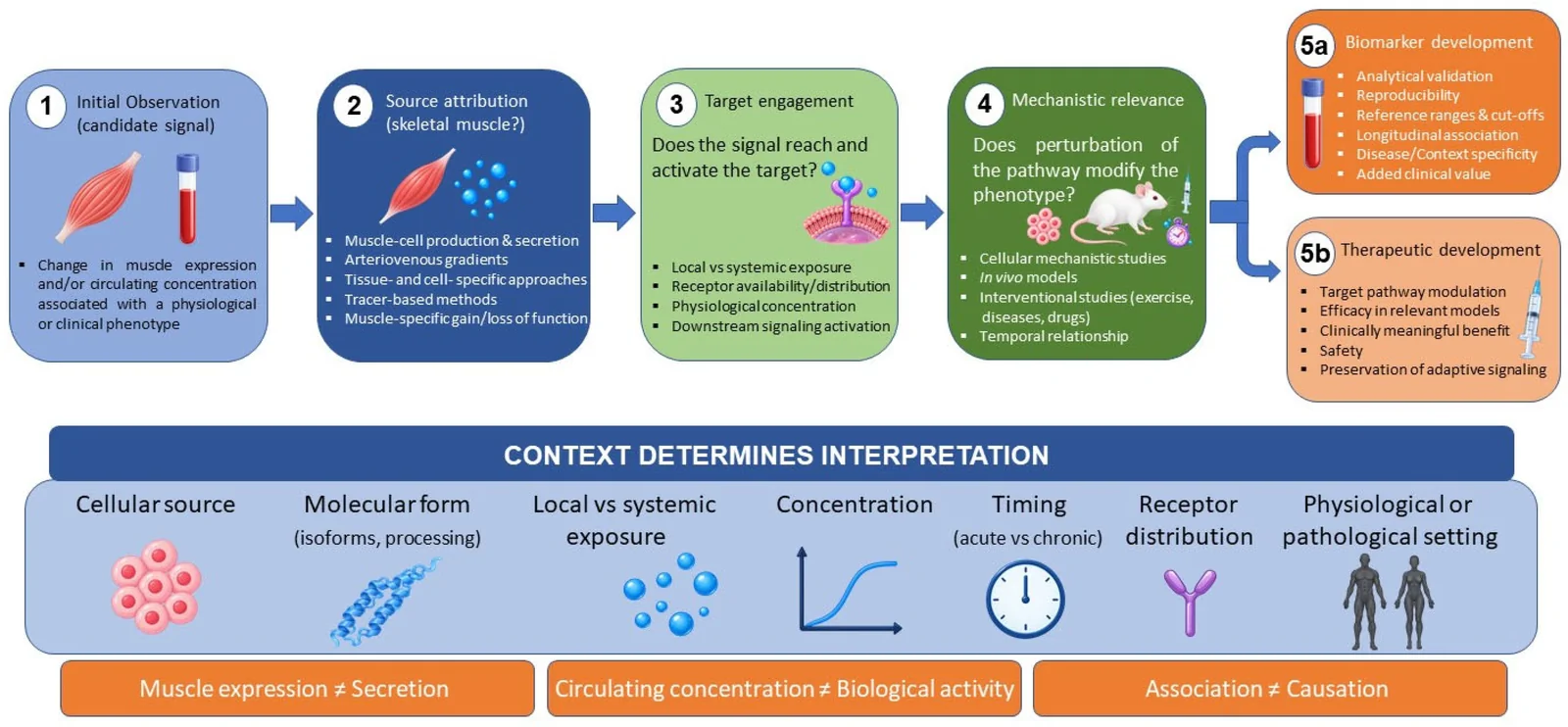
Stepwise framework for establishing the biological and translational relevance of candidate myokines. An initial observation linking muscle expression and/or circulating concentration of a candidate signal to a physiological or clinical phenotype should be followed by evidence establishing skeletal-muscle production and release, relevant target-tissue exposure, receptor engagement and downstream signaling, and mechanistic evidence that perturbation of the pathway modifies the associated phenotype. Candidates may subsequently progress toward biomarker development, requiring analytical validation, reproducibility, definition of reference ranges and cut-offs, longitudinal association, context specificity, and added clinical value, or toward therapeutic development, requiring evidence of effective pathway modulation, clinically meaningful benefit, acceptable safety, and preservation of physiologically adaptive signaling. Interpretation at every stage is influenced by cellular source, molecular form, local versus systemic exposure, concentration, timing, receptor distribution, and the physiological or pathological setting. Importantly, muscle expression should not be equated with secretion, circulating concentration with biological activity, or association with causation.

Taken together, the evidence reviewed here supports a conceptual shift from cataloguing molecules detected in muscle or blood toward defining the conditions under which a candidate myokine has physiological or pathological relevance. A stepwise framework integrating the principal requirements for this interpretation and for subsequent clinical translation is summarized in Figure 3. The designation “myokine” identifies a muscle-derived signaling origin rather than a homogeneous functional class. Its interpretation therefore requires separation of several distinct questions: whether skeletal muscle produces and releases the factor under the condition of interest; whether the signal reaches and engages biologically relevant target cells; whether receptor activation produces downstream functional consequences; and whether the observed change is causal, compensatory, secondary to tissue injury, or simply a marker of disease severity. These questions must be addressed within a defined temporal and disease-specific context, because acute local signaling during exercise, injury, or regeneration may differ fundamentally from sustained systemic exposure in chronic disease.

Future investigation should therefore move beyond isolated measurements of muscle expression or circulating concentration toward integrated source-attribution and mechanistic studies. Tissue- and cell-specific profiling, arteriovenous and tracer-based approaches, serial sampling, and direct assessment of receptor engagement and downstream pathway activation should be combined, where feasible, with longitudinal and interventional human studies. Progress toward biomarker application will require analytical validation, reproducibility, characterization of temporal and disease-specific variability, and demonstration of added clinical value. Progress toward therapeutic application will additionally require evidence that selective modulation of the relevant pathway produces clinically meaningful benefit with acceptable safety and without disrupting adaptive signaling in other tissues or physiological contexts.

From this perspective, the most informative future model of myokine biology is likely to be network-based rather than molecule-centered. Understanding how muscle-derived signals interact with one another and with signals originating from other tissues, and how cellular source, timing, receptor distribution, tissue exposure, and disease environment reshape these interactions, may allow the field to distinguish descriptive associations from mechanistically supported and clinically actionable pathways.

## Figures and Tables

**Table 1:** Summary of the Main Myokines and Muscle-Derived Factors: Sources, Actions, Signaling, Evidence, and Potential Clinical Relevance.

Myokine/Factor	Principal Muscle-Associated Source	Principal Biological Functions	Main Receptor/Signaling Pathway (s)	Evidence Represented in the Review	Potential Clinical Relevance
**Cytokines and Chemokines**					
**IL-6**	Contracting myofibers; satellite cells, macrophages, endothelial and stromal/fibro-adipogenic cells may contribute to injury or chronic inflammation.	Substrate metabolism; satellite-cell proliferation; load-induced hypertrophy; extracellular-matrix remodeling; early regeneration. Transient/local signaling is generally adaptive, whereas sustained systemic signaling may be catabolic.	IL-6Rα/gp130; classical and trans-signaling; mainly JAK/STAT3. AMPK contributes to metabolic actions.	Human arteriovenous studies demonstrate net release from exercising muscle; human metabolic data plus cellular and animal causal evidence for myogenic/regenerative actions.	Exercise and repair adaptation; chronic inflammatory/catabolic states and muscle wasting. Interpretation of elevated systemic IL-6 requires attention to non-muscle sources.
**IL-7**	Differentiated human myotubes and satellite-cell-derived myogenic cells.	Satellite-cell migration; restraint of terminal myogenic differentiation; possible muscle-immune communication.	IL-7Rα/CD127 + common γ-chain/CD132; JAK1/JAK3 → STAT5.	Human myotube production and training-related muscle mRNA responses are documented; functional evidence is mainly cellular. Net muscular release in humans has not been demonstrated.	Possible relevance to regeneration, aging/inflammation and muscle-immune communication, but physiological and clinical significance remains uncertain.
**IL-15**	Muscle fibers and cultured myogenic cells; IL-15 may be produced/presented as IL-15/IL-15Rα complexes.	Muscle growth and myogenic differentiation; regenerative environment; oxidative metabolism; adipose and immune communication; selected anti-wasting effects.	IL-15Rα with IL-2/IL-15Rβ (CD122) and γ-chain (CD132); trans-presentation; JAK1/JAK3 → STAT3/STAT5; PI3K/Akt and MAPK; AMPK in metabolic effects.	Extensive cellular and animal evidence, including gain/loss-of-function models; human myogenic and exercise data exist, but physiological endocrine release and systemic effects remain less firmly established.	Potential relevance to muscle wasting, metabolic regulation and regeneration; may also enhance cytotoxic immune responses in inflammatory myopathy. Therapeutic translation and safety remain uncertain.
**IL-1β**	Myoblasts and myotubes under inflammatory/metabolic stress; macrophages and other resident/infiltrating cells may contribute substantially in injured muscle.	Early satellite-cell/progenitor expansion and repair when transient; excessive or prolonged signaling can promote muscle catabolism.	Inflammasome/caspase-1 processing of pro-IL-1β; IL-1R1 → NF-κB and MAPK pathways.	Myogenic production is demonstrated mainly in cellular systems, with experimental evidence for injury/repair responses; muscle-cell-specific human evidence is limited in the reviewed data.	Inflammatory and metabolic muscle stress; balance between regenerative inflammation and catabolic signaling.
**TNF-α**	Myoblasts/myotubes under selected inflammatory and myogenic conditions; macrophages and other immune/stromal cells are often predominant in injured or diseased muscle.	Transient low-level signaling can support p38-dependent differentiation and regeneration; sustained exposure suppresses myogenesis, promotes protein loss and impairs insulin signaling.	TNFR1/TNFR2; NF-κB, MAPK/p38, JNK and reactive-oxygen-species-dependent mechanisms.	Cellular and animal causal evidence; human infusion studies demonstrate impaired muscle insulin signaling, and human COPD muscle associations are reported. Physiological exercise does not appear to cause substantial muscular TNF-α release.	Inflammatory muscle remodeling, insulin resistance and wasting; possible relevance in chronic systemic disease and COPD, with strong dependence on source, dose and duration.
**LIF**	Contracting skeletal muscle/myotubes; primary human myotubes can produce and secrete LIF.	Myoblast proliferation; regulation of expansion versus differentiation; muscle regeneration; contribution to load-induced hypertrophy.	LIF receptor + gp130; mainly JAK/STAT3, with MAPK/ERK and PI3K/Akt.	Human muscle mRNA and myotube secretion are documented; causal evidence for regeneration and hypertrophy is mainly from animal models. Circulating LIF may remain undetectable after exercise.	Local muscle adaptation and repair; timing- and concentration-dependent effects. Human clinical relevance remains to be established.
**CXCL8** **(IL-8)**	Myofibers/myotubes; endothelial cells, fibroblasts, macrophages and infiltrating leukocytes can also contribute within muscle.	Endothelial survival/migration and angiogenesis; extracellular-matrix remodeling; neutrophil recruitment; possible direct myogenic/anticatabolic effects.	CXCR1/CXCR2 GPCRs; MAPK/ERK and PI3K/Akt pathways.	Human myotube secretion and muscle expression are demonstrated; mechanistic evidence is largely cellular/animal, and the specific human contribution to exercise-induced angiogenesis is less conclusive than for VEGF-A.	Vascular adaptation and injury inflammation; excessive signaling has been linked experimentally to vascular dysfunction and muscle catabolism in pathological settings.
**CCL2** **(MCP-1)**	Injured/regenerating myofibers plus mononuclear, endothelial, fibro-adipogenic and other stromal/inflammatory cells.	CCR2+ monocyte/macrophage recruitment; tissue clearance and trophic support of regeneration; possible myogenic-cell migration/proliferation. Sustained signaling may drive pathological remodeling.	CCR2 GPCR; intracellular calcium mobilization, cytoskeletal remodeling, migration, proliferation and survival pathways.	Human muscle expression after exercise is documented; strong causal injury/regeneration evidence is from animal models, with direct myogenic effects mainly cellular/animal. Net release from exercising human muscle has not been directly established.	Muscle injury and recovery; persistent activation may contribute to fibrosis, fatty replacement and neuromuscular damage, including experimental ALS-related denervation.
**CXCL1 (GROα)**	Contracting murine and human myotubes; skeletal muscle and liver both respond to exercise, with hepatic CXCL1 partly regulated by muscle-derived IL-6.	Myogenic-cell calcium signaling/migration; neutrophil recruitment and early response to denervation; possible enhancement of fatty-acid oxidation and systemic metabolic effects.	CXCR2 GPCR; Gαi-dependent calcium signaling and downstream pathways controlling migration, proliferation and survival.	Human myotube secretion is demonstrated, whereas most causal functional and metabolic evidence is from animal models. Net release/endocrine relevance in exercising humans remains limited.	Stress/injury adaptation and denervation response; potential metabolic and inter-organ effects remain uncertain in humans.
**CXCL10** **(IP-10)**	Myotubes/myogenic cells; production is strongly induced by inflammatory signals and may be reduced by contraction in experimental systems.	CXCR3-dependent recruitment/retention of activated T cells; amplification of Th1 inflammation; anti-angiogenic effects; possible myogenic differentiation and connective-tissue signaling.	CXCR3; chemotaxis/cytoskeletal signaling. Production is regulated by STAT1 (IFN-γ) and NF-κB (TNF-α); functional redundancy with CXCL9/CXCL11.	Human myogenic-cell and post-damaging-exercise observations are available; mechanistic and loss-of-function evidence is mainly cellular/animal. Proposed vascular/endocrine actions remain unconfirmed in humans.	Inflammatory myopathies and chronic local inflammation; possible restraint of vascular adaptation. Muscle-skin and metabolic-disease implications remain preliminary.
**Growth Factors and Growth-related proteins**					
**IGF-1/IGF-1R**	Locally produced skeletal-muscle IGF-1 acting on myofibers, satellite cells and myoblasts; circulating endocrine IGF-1 is largely not muscle-specific.	Protein synthesis and hypertrophy; inhibition of atrophy-associated signaling; satellite-cell activation/proliferation and later differentiation; regeneration; modulation of inflammation and fibrosis.	IGF-1 receptor tyrosine kinase; PI3K/Akt → mTOR; GSK-3 inhibition; Akt-mediated FoxO inhibition.	Extensive cellular and animal causal evidence, including muscle-restricted transgenic models; human muscle splice-variant/exercise regulation is documented. Translation of overexpression results depends on isoform, exposure and tissue restriction.	Muscle growth and repair; potential relevance to aging, disuse, neuromuscular disease and cachexia. Local versus systemic delivery and isoform-specific effects require caution.
**MGF/** **IGF-1Ec**	Mechanically responsive IGF1Ec transcript/pro-IGF-1 isoform expressed in skeletal muscle; independent secretion of an Ec-domain peptide is unproven.	Associated with mechanical loading, satellite-cell activation and muscle repair; biological effects independent of mature IGF-1 remain controversial.	No independent receptor/signaling pathway has been established for a physiologically secreted Ec-domain peptide.	Evidence principally concerns transcript/pro-IGF-1 regulation. Human exercise and COPD muscle expression data exist, but independent peptide production/secretion has not been demonstrated.	Potential marker of mechanically induced growth/repair programs; independent therapeutic or endocrine relevance remains unestablished.
**HGF**	Extracellular-matrix-bound HGF in adult muscle; differentiated myotubes and satellite-cell cultures can produce HGF locally. Relative in vivo cellular contributions remain uncertain.	Activation of quiescent satellite cells; myogenic proliferation, migration, stage-dependent differentiation and fusion; modulation of reparative macrophage responses.	MET/c-Met receptor tyrosine kinase; MAPK/ERK and PI3K/Akt. Mechanical release involves Ca^2+^/calmodulin, nitric oxide and matrix metalloproteinases; macrophage effects involve CaMKKβ/AMPK.	Cellular and animal studies provide most mechanistic/causal evidence; local bioactive HGF production is demonstrated, but the precise in vivo cellular source and human physiological contribution are less clearly defined.	Early muscle repair and regeneration; relevance is primarily local and experimental rather than as a conventional circulating endocrine myokine.
**VEGF-A**	Skeletal muscle fibers; released into the muscle interstitium in response to contraction, hypoxia and increased metabolic demand.	Endothelial-cell survival, proliferation and migration; maintenance of capillarity; exercise/training-induced angiogenesis.	VEGFR2 on endothelial cells; pro-angiogenic signaling downstream of receptor activation.	Strong experimental evidence, including myocyte-specific deletion showing reduced capillarity and impaired training adaptation; muscle expression/release is well established.	Microvascular adaptation and exercise capacity; insufficient muscle-derived VEGF-A may contribute to reduced capillarity and impaired training responses.
**FGF21**	Skeletal muscle under mitochondrial/metabolic stress; expression is low in healthy resting muscle. Liver is the principal source of circulating FGF21 under most physiological conditions.	Mitophagy and mitochondrial quality control; muscle-mass regulation; adipose-tissue remodeling/browning; neuromuscular-junction changes under denervation stress.	FGFR (especially FGFR1c) + β-Klotho → ERK1/2; muscle expression is linked to integrated stress-response pathways including eIF2α/ATF4.	Mechanistic evidence is predominantly cellular and animal. Human arteriovenous studies during exercise show splanchnic secretion without detectable net release from the exercising leg.	Marker/mediator of mitochondrial and metabolic stress; chronic muscular FGF21 may contribute to fasting/denervation-associated wasting. Muscle-specific endocrine relevance in humans is limited.
**GDF15**	Stress-induced skeletal muscle cells/myotubes; in injured muscle, reparative macrophages can be a major local source. Multiple non-muscle tissues also produce GDF15.	Systemic stress signaling to the brain; regulation of food intake, energy balance and adipose metabolism; possible lipid mobilization; regenerative inflammation and progenitor proliferation in injured muscle.	GFRAL + RET in hindbrain neurons for canonical endocrine signaling; canonical receptor is not established in normal skeletal muscle. Proposed peripheral actions may be GFRAL-independent.	Human primary myotube secretion and exercise-induced muscle mRNA are documented; strong causal systemic evidence comes from animal models. Net muscular contribution to circulating GDF15 in physiological exercise remains uncertain.	Potential marker/mediator of muscular mitochondrial stress and systemic metabolic adaptation; relevance to appetite/energy balance and regenerative inflammation, with major tissue-source caveats.
**Myostatin (GDF-8)**	Developing and mature skeletal muscle fibers; predominantly local autocrine/paracrine production, with circulating ligand often latent or inhibitor-bound.	Physiological restraint of muscle growth; inhibition of myoblast/satellite-cell activation and differentiation; suppression of Akt-mTOR anabolism; limitation of hypertrophy; promotion of fibroblast activity and ECM deposition.	ACVR2B/ACVR2A + ALK4/ALK5 → SMAD2/3-SMAD4; crosstalk with MAPK, PI3K/Akt and mTOR.	Extensive cellular and animal causal evidence plus human genetic evidence; human exercise studies show variable *MSTN* expression. Circulating total protein does not necessarily reflect active local signaling.	Major therapeutic target for muscle wasting, sarcopenia and neuromuscular disease; inhibition can increase muscle mass, but gains in mass do not necessarily translate into proportional functional benefit and receptor-level approaches may lack specificity.
**Peptides, Neurotrophic factors and Extracellular-matrix-associated proteins**					
**Irisin**	Differentiated skeletal muscle cells/myotubes via proteolytic processing of FNDC5; other tissues also express FNDC5.	Myogenic differentiation/fusion; satellite-cell activation; anabolic/hypertrophic and regenerative effects in experimental models; glucose uptake; proposed adipose browning/endocrine actions.	αV integrins, especially αVβ5; FAK/AKT/mTOR, ERK1/2, PI3K/Akt/mTOR and AMPK; may interact with IL-6-related signaling.	Extensive cellular and animal evidence; human muscle-cell secretion and plasma presence by mass spectrometry are documented, but physiological functional effects in humans remain less firm and immunoassay results are inconsistent.	Potential metabolic, regenerative and anti-wasting relevance; proposed biomarker roles in metabolic disease/sarcopenia and adipose browning require caution because of assay, dose and tissue-source limitations.
**Apelin**	Skeletal muscle cells; also produced by adipose, cardiovascular and other tissues.	Mitochondrial biogenesis/homeostasis; autophagy; anti-inflammatory effects; muscle stem-cell regeneration; maintenance of muscle function and performance.	APLNR/APJ G-protein-coupled receptor; downstream pathways are not detailed in the present Review.	Human skeletal-muscle expression increases with endurance training; most causal evidence for muscle function, aging and regeneration derives from animal models.	Age-related muscle dysfunction and sarcopenia are potential areas of relevance; restoration of apelin-APLNR signaling is therapeutically interesting but efficacy/safety in older humans is unestablished.
**Musclin (Osteocrin)**	Predominantly skeletal muscle and bone; muscle expression increases with physical activity.	Potentiation of natriuretic-peptide signaling; mitochondrial biogenesis and oxidative capacity; exercise endurance; possible cardiovascular protection.	Binds NPR-C and reduces natriuretic-peptide clearance → enhanced cGMP signaling; activity-induced expression involves Ca^2+^-dependent Akt1 relief of FoxO1 repression.	Causal evidence is mainly experimental/animal (loss-of-function and recombinant rescue); contribution to exercise adaptation and metabolic regulation in humans remains incompletely established.	Exercise tolerance and metabolic adaptation; possible cardiovascular protection during pathological overload, supported mainly by animal models.
**BDNF**	Skeletal muscle fibers, myoblasts and satellite cells; molecular form is important (proBDNF versus mature BDNF).	Lipid oxidation/metabolic adaptation; regulation of satellite-cell proliferation/differentiation; early muscle regeneration.	Mature BDNF → TrkB; proBDNF → p75NTR-containing complexes. BDNF can activate AMPK and inhibit acetyl-CoA carboxylase in muscle models.	Human muscle expression is exercise-responsive, and arteriovenous data support release of proBDNF (not mature BDNF) after high-intensity exercise; causal regenerative evidence is largely cellular/animal.	Metabolic adaptation and muscle repair; clinical interpretation requires distinction between proBDNF and mature BDNF and recognition that circulating mature BDNF is not clearly muscle-derived.
**FSTL1**	Primary human skeletal muscle cells/myotubes; heart, vascular cells, adipose tissue and other organs are also sources.	Endothelial survival/migration, angiogenesis and revascularization; skeletal-muscle-to-cardiovascular communication; direct myogenic roles are less well established.	Akt/eNOS/nitric oxide signaling in endothelial cells; AMPK-dependent regulation of vascular smooth-muscle responses.	Human muscle-cell secretion and exercise-associated circulating changes are described; strongest causal vascular evidence is from experimental/animal muscle-specific manipulation. Direct myogenic effects remain insufficiently established.	Vascular adaptation, ischemic revascularization and cardiovascular protection are the main potential areas; source attribution is important for systemic effects.
**Decorin**	Skeletal muscle cells and extracellular matrix; secretion increases with contraction. Other tissues also contribute to circulating decorin.	Attenuation of myostatin activity; linking ECM remodeling to muscle growth; possible support of myogenic growth/differentiation.	Direct binding to mature myostatin (Zn^2+^-dependent), reducing myostatin inhibitory activity; no separate canonical receptor pathway is established in this Review.	Muscle-cell/exercise-associated production is documented; mechanistic evidence for myostatin antagonism and growth effects is mainly experimental.	Potential relevance to hypertrophy and ECM remodeling through modulation of myostatin; direct regenerative and clinical effects remain less firmly established.
**METRNL**	Skeletal muscle, adipose tissue and immune cells; macrophages are a major functionally relevant source in injured muscle.	Promotion of reparative macrophage phenotype and macrophage-derived IGF-1-dependent regeneration; adipose thermogenesis, energy expenditure and glucose homeostasis in experimental models.	Specific receptor is not defined in the Review; regenerative effects involve STAT3 activation in macrophages → IGF-1 signaling to satellite cells.	Human exercise studies mainly show muscle mRNA regulation; direct net muscular secretion has not been established. Most causal regenerative/metabolic evidence is from animal models.	Potential regenerative and metabolic relevance; attribution to myofibers is particularly uncertain in injured muscle, and human systemic relevance remains incompletely established.

**Note:** The evidence column summarizes the type and level of evidence described in the Review and is not intended as a formal grading system. Human evidence may refer to muscle expression/secretion, physiological studies, interventions, or clinical associations and does not necessarily establish tissue-specific causality. Potential clinical relevance is stated cautiously and does not imply validated biomarker or therapeutic use. See the main text for references and detailed limitations. ACVR, activin receptor; Akt, protein kinase B; AMPK, AMP-activated protein kinase; APLNR, apelin receptor; CCL, C-C motif chemokine ligand; CXCL/CXCR, C-X-C motif chemokine ligand/receptor; ECM, extracellular matrix; eNOS, endothelial nitric oxide synthase; FGF/FGFR, fibroblast growth factor/receptor; GDF, growth differentiation factor; GFRAL, GDNF family receptor α-like; HGF, hepatocyte growth factor; IGF-1/IGF-1R, insulin-like growth factor 1/receptor; IL, interleukin; JAK/STAT, Janus kinase/signal transducer and activator of transcription; LIF, leukemia inhibitory factor; MAPK/ERK, mitogen-activated protein kinase/extracellular signal-regulated kinase; MET, hepatocyte growth factor receptor; METRNL, meteorin-like; MGF, mechano growth factor; mTOR, mechanistic target of rapamycin; NF-κB, nuclear factor κB; NPR, natriuretic peptide receptor; PI3K, phosphoinositide 3-kinase; RET, receptor tyrosine kinase RET; SMAD, SMAD family proteins; TNF, tumor necrosis factor; VEGF/VEGFR, vascular endothelial growth factor/receptor.

## Data Availability

No new data were created or analyzed in this study. Data sharing is not applicable to this article.
